# Yellowfin tuna (*Thunnus albacares*) foraging habitat and trophic position in the Gulf of Mexico based on intrinsic isotope tracers

**DOI:** 10.1371/journal.pone.0246082

**Published:** 2021-02-24

**Authors:** Meliza Le-Alvarado, Alfonsina E. Romo-Curiel, Oscar Sosa-Nishizaki, Oscar Hernández-Sánchez, Leticia Barbero, Sharon Z. Herzka

**Affiliations:** 1 Department of Biological Oceanography, Center for Scientific Research and Higher Education of Ensenada (CICESE), Ensenada, Baja California, México; 2 Ocean Chemistry and Ecosystems Division, Cooperative Institute for Marine and Atmospheric Studies (CIMAS), NOAA’s Atlantic Oceanographic and Meteorological Laboratory, Miami, Florida, United States of America; Universidad de Cádiz, Facultad de Ciencias del Mar y Ambientales, SPAIN

## Abstract

Yellowfin tuna (YFT, *Thunnus albacares*) is a commercially important species targeted by fisheries in the Gulf of Mexico (GM). Previous studies suggest a high degree of residency in the northern GM, although part of the population performs movements to southern Mexican waters. Whether YFT caught in southern waters also exhibit residency or migrate to the northern gulf is currently uncertain, and little is known regarding their trophic ecology. The isotopic composition (bulk & amino acids) of YFT muscle and liver tissues were compared to a zooplankton-based synoptic isoscape from the entire GM to infer feeding areas and estimate Trophic Position (TP). The spatial distribution of δ^15^N_bulk_ and δ^15^N_Phe_ values of zooplankton indicated two distinct isotopic baselines: one with higher values in the northern GM likely driven by denitrification over the continental shelf, and another in the central-southern gulf, where nitrogen fixation predominates. Based on the contribution of the two regional isotopic baselines to YFT tissues, broad feeding areas were inferred, with a greater contribution of the northern GM (over a one-year time scale by muscle), and to a lesser extent in the central-southern GM (over the ca. 6-month scale by liver). This was corroborated by similarities in δ^15^N_Phe_ values between YFT and the northern GM. TP estimates were calculated based on stable isotope analysis of bulk (SIA) and compound-specific isotope analysis (CSIA-AA) of the canonical source and trophic amino acids. Mean TP based on SIA was 4.9 ± 1.0 and mean TP based on CSIA-A was 3.9 ± 0.2. YFT caught within the Mexican region seem to feed in northern and in central and southern GM, while feeding in the northern GM has a temporal component. Thus, management strategies need to consider that YFT caught in US and Mexican waters are a shared binational resource that exhibit feeding migrations within the GM.

## Introduction

**Fig 1:**
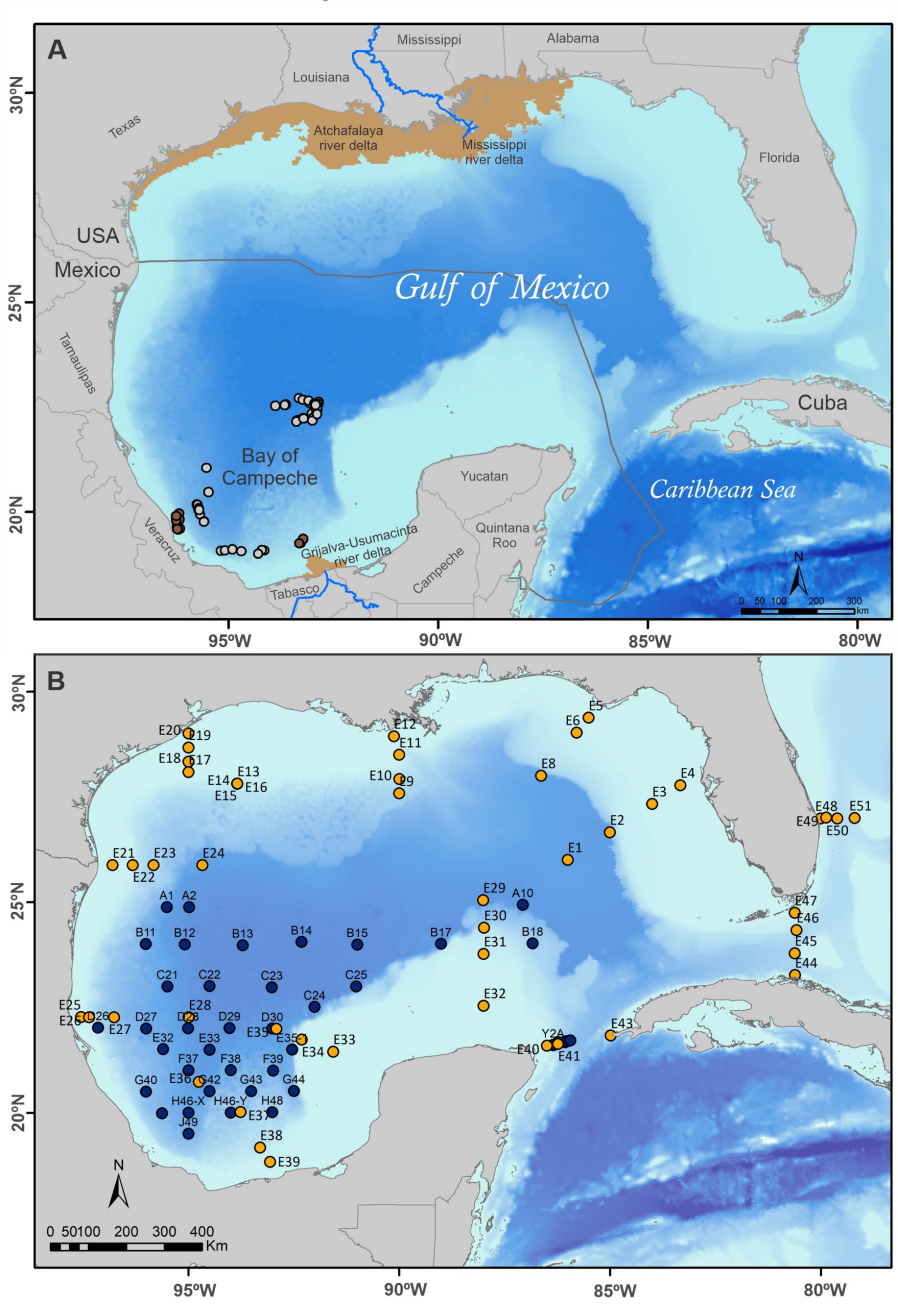
Location of sample collection sites in the Gulf of Mexico. (A) Sampling location of yellowfin tuna (*Thunnus albacares*) in the Bay of Campeche (brown dots, 2017; n = 14) and the Bay of Campeche and northwest of the Yucatan shelf (light grey dots, 2018; n = 58). The brown shaded areas illustrate higher surface chlorophyll mean concentrations associated with the Atchafalaya-Mississippi, and Grijalva-Usumacinta river systems during the zooplankton sampling period. (B) Location of zooplankton sampling stations in the Gulf of Mexico. Stations sampled during the GOMECC-3 cruise (July- August, 2017) are indicated with orange dots, and stations covered during the XIXIMI-06 cruise (August-September, 2017) are indicated in dark blue dots. Bathymetry, chlorophyll-a means, rivers, and country limits used to build maps were downloaded from free licensed data bases available at: https://www.ngdc.noaa.gov/mgg/ibcca/, https://www.inegi.org.mx/app/mapas/, https://oceancolor.gsfc.nasa.gov/l3/, http://www.cec.org/es/atlas-ambiental-de-america-del-norte/lagos-y-rios-2009/, http://www.diva-gis.org/gdata.

Yellowfin tuna (YFT), *Thunnus albacares*, is a highly valuable resource that is fished worldwide. It constitutes the second-largest tuna fishery in the world and represents a quarter of the total catch globally [1]. In the Atlantic Ocean, YFT is the second most important species supporting commercial and recreational fisheries, and it is currently managed as a single stock by the International Commission for the Conservation of Atlantic Tunas [2]. In the Gulf of Mexico (GM) and the Caribbean Sea, the fishery is carried out year-round, with a maximum catch during the summer [3]. In the southern GM, within the Mexican Exclusive Economic Zone (Fig 1A), it is the main targeted oceanic species by the local pelagic longline fishery which is active year-round with higher captures during summer months [4, 5]. YFT is classified as near-threatened by the International Union for Conservation of Nature, and according to the most recent stock assessment, the Atlantic Ocean stock is not overfished [2]. Therefore, it is relevant to characterize their habitat use, migration patterns, and feeding ecology in order to implement effective management strategies policies for this multi-national fishing resource and continue with the current status.

Some YFT perform extensive transoceanic migrations that are mainly associated with spawning and feeding. Although the specific migration routes remain unclear, YFT do serve as ecological links and means of energy transfer between ocean basins [6–8]. According to several studies, four spawning areas are recognized within the Atlantic Ocean: two in the eastern Atlantic (Cape Verde and the Gulf of Guinea) and two in the western Atlantic (the GM and the Caribbean Sea) [9–11]. In the GM the spawning season occurs between May and August, as indicated by the presence of spawning females and high larval abundance in the northern gulf [11–12]. Recently, the natal origin of adults and subadults of YFT captured in the northern GM were determined based on otolith microchemistry (δ^13^C and δ^18^O analysis and trace elements). Half of the YFT analyzed are originated in the GM and appear to be permanent residents, while the rest were inferred to come from the eastern Atlantic [13, 14]. Hence, different subpopulations with specific migration patterns may exist throughout the Atlantic Ocean.

In the northern GM, YFT seem to have some degree of residency, although part of the population migrates to the central and southern GM seasonally [15–18]. Rooker et al. [19] tracked the horizontal movements of 54 individuals tagged with pop-up archival satellite tags over 6 to 12 months in the northern shelf waters of the GM during 2008–2016, and found limited spatial displacement within the U.S. Exclusive Economic Zone. Nonetheless, a few individuals did migrate to Mexican waters during fall and winter (see also Hoolihan et al. [17]). However, the lack of long-term (> 12 months) and system-wide tracking resulted in a limited characterization of the full range of migratory pathways within the GM over the annual cycle [19]. In the central southern GM, the YFT Mexican fishery register captures year-round within the Mexican Exclusive Economic Zone, indicating that YFT are present throughout the year and likely performing movements within the basin to meet breeding and foraging requirements [4, 5].

Tunas invest much of their energy in the search for food in oligotrophic pelagic habitats, where food availability is scarce [20, 21]. YFT is an opportunistic predator with a generalist foraging strategy, feeding on a wide variety and size of pelagic prey found in warmer surface waters [22–24]. The habitat preferences of YFT are linked to high prey densities that occur in association with specific oceanographic features such as fronts, eddies, and steep bathymetry (i.e., near continental slopes and seamounts), where phytoplankton production is enhanced and therefore, higher concentrations of prey occur [15, 16, 22]. Hence, spatial variation in food web structure and availability are likely to influence YFT tuna distribution and foraging.

During the last 50 years, fishery landings in the northern hemisphere have changed from large piscivorous fish to fish species that are planktivorous or that feed on small invertebrates, which implies drastic changes in the structure of marine trophic food webs [25–27]. One way to characterize these changes is by examining the trophic position (TP) of top predators since they integrate the energy flow within the ecosystem in which they forage [28]. The TP of top predators, such as YFT from the GM, provide an indicator of the integrity and health of ecosystems [29], and thus must be documented over the long term. Currently, there is limited knowledge regarding the foraging ecology, and TP of YFT caught in the central and southern GM.

TP has been traditionally assessed through stomach content analysis. However, this analysis only allows for inferences regarding the most recently ingested diets and is labor intensive due to the need for taxonomic identification of prey sampled from the stomachs of many individuals (i.e., Hyslop [30]). Biochemical intrinsic tracers such as bulk stable isotopes analysis (SIA) and compound-specific stable isotope analysis of amino acids (CSIA-AA) are complementary approaches to stomach content analysis because they provide information on TP that is integrated over time.

SIA of bulk tissues, particularly of carbon (δ^13^C) and nitrogen (δ^15^N), have been used to elucidate migratory pathways and habitat use patterns [31–33]; both elements reflect the isotopic composition of the assimilated diet in metabolically active consumer tissues [34]. The integration time of a tissue’s isotopic composition depends on the rate of isotopic turnover, which is largely a function of its metabolic activity, as well as an individual’s life stage and growth rates [33, 35, 36]. Isotope discrimination leading to enrichment in ^15^N or ^13^C in consumer tissues relative to assimilated prey [37, 38] is commonly reported as the trophic enrichment factor (TEF) [39, 40]. Because bulk δ^15^N values become consistently enriched in ^15^N with each trophic level, it allows for estimates of TP if the isotopic composition at the base of the food web (the isotopic baseline) is adequately characterized [32, 41].

In addition, since consumers reflect the isotopic composition at the base of the food web, bulk tissue SIA and CSIA-AA can yield insight into the migration patterns of organisms that feed in areas differing in baseline isotopic composition [42, 43]. Regional biochemical processes cause spatial differences in the isotopic composition of nutrient sources and primary producers, resulting in isotopic gradients that can be used to infer feeding habitat and migratory pathways [44, 45]. Carbon baseline values reflect the isotopic composition of primary producers and the dissolved inorganic carbon pool [46], whereas nitrogen depends mainly on nitrogen sources and the regionally predominant biogeochemical process such as N fixation and denitrification [47]. The spatial distribution of isotopic values, depicted as an *isoscape*, is often constructed using the isotopic composition of primary consumers (e.g., zooplankton, benthic filter feeders), since they tend to smooth out the temporal variability exhibited by producers, particularly phytoplankton [45, 48]. By serving as the spatial reference of the isotopic baseline, isoscapes can be used to infer feeding habitats and movement of animals over various spatial scales [44, 45]. If consumer δ^15^N values are consistent with those of a local baseline, then an individual can be considered a resident that has partially or fully equilibrated to the isotopic composition of local prey, whereas recent immigrants will exhibit distinct isotopic values [44, 49]. A correct characterization of the isotopic baseline is crucial, because otherwise the interpretation of migration patterns, diet shifts, or both can be confounded, and the TP of a consumer can be under or over-estimated [50–52].

Compound-specific analysis of amino acids (CSIA-AA) is a complementary approach to bulk SIA for estimating TP and inferring foraging ecology [53–55], with the advantage that the isotopic baseline and TP can be inferred from a single tissue sample [43, 56]. AAs that exhibit little or no discrimination, known as source AAs, reflect the isotopic baseline as synthesized by primary producers and assimilated by consumers. Phenylalanine (Phe) shows low and somewhat consistent TEFs in a diverse array of consumer-diet relationships and is considered the canonical source AA (reviewed by O’Connell [57]). For this reason, several studies have used δ^15^N_Phe_ values to estimate δ^15^N_baseline_ successfully [48, 58, 59]. AA with high isotope discrimination are considered trophic AAs [53], and reflect a consumer’s trophic position. Glutamic acid (Glu) shows high and relatively consistent isotope discrimination and is considered the canonical trophic AA [57, 59]. Based on the isotopic composition of source and trophic AAs, and empirical estimates of TEFs, the TP of consumers can be estimated [42, 56].

Here, the foraging habitat and TP of YFT caught in the south-central GM is inferred based on both bulk δ^13^C and δ^15^N values and the δ^15^N values of Phe and Glu measured in muscle and liver tissues, which integrate different feeding periods. We hypothesized that the isotopic composition of YFT captured in the south-central GM would be indicative of feeding habitat in that region. A zooplankton-based synoptic isoscape was generated for the entire GM, resulting in the characterization of two distinct regional isotopic baselines. A two-source Bayesian mixing model was applied to estimate the relative contribution of each baseline to YFT tissues to infer foraging habitat, and TP was assessed with bulk SIA considering both regional baselines and CSIA-AA.

## Methods

The government of Mexico allowed for research within their Exclusive Economic Zones during the GOMECC-3 cruise (PPFE/DGOPA-137/17, EG0082017). No permits are required for sampling of zooplankton in Mexican waters (XIXIMI-06 cruise). Yellowfin tuna were collected during permitted commercial fishing operations within the Mexican EEZ.

### Study area

The GM is a semi-enclosed basin located in the western Atlantic with a maximum width of 1,500 km (18 to 30°N, 82 to 98°W). One of the major drivers of mesoscale circulation in the GM is the Loop Current, which penetrates the GM through the Yucatan Strait. The northern GM shelf is heavily influenced by the inflow of the Mississippi River system that discharges freshwater and sediments to the gulf [60]. Local wind stress and tidal currents provide forcing mechanisms for the mixing of freshwater and seawater, enhancing primary and secondary production in the region [61].

In the southern GM, the Bay of Campeche (south of 22°N) is a semi-closed region that encompasses the deep water region as well as the continental shelves of the states of Veracruz, Tabasco, and Campeche in Mexico (Fig 1A). The bay is characterized by a semi-permanent cyclonic gyre in its southwestern reaches, within which upwelled nutrient-rich waters sustains phytoplankton production that supports high prey biomass for top predators [62, 63]. The productivity of the continental shelves in the southern Bay of Campeche is strongly influenced by the freshwater discharge of the Grijalva-Usumacinta river system, which also increases regional productivity [64]. The Campeche Bank (or Yucatan shelf), located east of the Bay of Campeche, receives nutrient-rich water throughout the year due to regional upwellings [65, 66].

### Sample collection

YFT samples were collected during the summers of 2017 and 2018 (n = 72). The 2017 sampling (June 12 to July 2) took place on board the longline fishing vessel "Skypjack", and 14 tunas were captured within the Bay of Campeche (Fig 1A). In 2018 (August 7–20), 58 tunas were sampled within the Bay of Campeche and northwest of the Yucatan shelf on board the longline fishing vessel "O-toro". Tuna were measured for curved fork length (cm), and ~3 cm^3^ samples of white muscle (hereafter muscle) and liver were dissected [67]. The muscle was extracted from the central epaxial area dorsal to the ocular cavities to preserve the integrity of tuna destined for the commercialization of high-quality fillets. Both tissue samples were placed in labeled plastic bags and frozen at -20°C for transport to the laboratory and subsequent isotope analysis.

Zooplankton were collected throughout the GM during two concurrent oceanographic cruises held during the summer of 2017. The XIXIMI-06 cruise, conducted by CIGoM (Consorcio de Investigación del Golfo de México), was held from August 18 to September 10 and covered the deep water region of Mexico’s Exclusive Economic Zone (Fig 1B). The Gulf of Mexico Ecosystems and Carbon Cycle 2017 Cruise (GOMECC-3) was held from July 20 to August 20 by the NOAA (National Oceanic and Atmospheric Administration). GOMECC-3 covered stations that ran along nine transects within the gulf as well as the Yucatan Channel, Florida Straits, and Bahamas Channel [68]. A total of 44 and 55 stations were covered during the XIXIMI-06 and GOMECC-3 cruises, respectively, for a total of 95 stations.

Zooplankton collections were identical on both cruises. At each station, oblique tows to 200 m (depth permitting) were performed with 60 cm bongo nets fitted with 335 μm mesh nets. Twenty percent by volume of one of the net samples was separated for subsequent zooplankton isotope analysis by gaging to 500 ml, swirling, and withdrawing two 50 ml subsamples with a Hensel-Stempel pipette. Samples were frozen immediately in WhirlPack bags without preservatives. Zooplankton community composition was dominated by copepods, chaetognaths, ostracods, rhizarians, and polychaetes.

### Bulk stable isotope and CSIA-AA analyses

YFT muscle and liver tissue samples were thawed and rinsed with distilled water. From each sample, a small ~1 cm^3^ portion was extracted, placed in aluminum trays and dried in a Fisher Scientific® drying oven at 60°C for 48 hours. Dried samples were ground using an agate mortar to a fine homogeneous powder. For bulk SIA, a 0.8–1.2 mg subsample was weighed on an analytical balance, packaged in 5x9 mm Costech® tin capsules and stored in plastic trays. For δ^15^N CSIA-AA, a subset of muscle and liver tissues from 36 tunas (from all the samples collected in 2017 plus 22 samples chosen randomly from 2018) was selected. A subsample of 7–10 mg was weighed and stored in pre-combusted 5 ml glass vials with a plastic cap.

Zooplankton samples were thawed, and size fractions of <1000 μm and >2000 μm were separated with a NITEX sieve, dried, and processed as described above. Bulk δ^13^C and δ^15^N values were analyzed on the smaller size fraction for all zooplankton samples. For CSIA-AA, a subset of 22 samples of zooplankton >2000 μm were analyzed; stations were chosen based on a preliminary analysis of the spatial distribution of zooplankton bulk δ^15^N values. For some stations, the minimum weight required for CSIA-AA analysis was not obtained, and samples from neighboring stations with similar bulk δ^15^N values were combined and homogenized.

Carbon and nitrogen SIA and nitrogen CSIA-AA analysis were performed at the Stable Isotope Facility of the University of California at Davis, U.S.A. For SIA, samples were analyzed using a PDZ Europe ANCA-GSL elemental analyzer with an interface to a PDZ Europe 20- δ^15^N 20 ratio mass spectrometer (Sercon Ltd., Cheshire, UK). Methodology applied for the CSIA-AA analysis is as described in Yarnes & Herszage [69]. The standard deviations (SD) of the laboratory’s quality assurance material (bovine liver) for SIA were 0.02‰ for δ^15^N and ≤0.03‰ for δ^13^C. The standard deviation of individual AA isotope ratios from multiple (usually 2) injections of single samples for zooplankton, muscle, and liver samples were 0.5‰ and 0.4‰ for Phe and Glu, respectively.

The isotopic composition of the tissue and individual AA values are reported in delta (δ) notation relative to Vienna PeeDee Belemnite for δ^13^C and atmospheric nitrogen for δ^15^N [70] using the following Eq (1):

$$\delta \;X\;(‰)=\left[(\frac{R_{sample}}{R_{standard}}-1)\right]\times 1000$$ (1)

where *X* is either ^13^C or ^15^N, and *R* is the relative abundance of the heavy to light isotope ratio of the sample or standard. Isotopic values are expressed in *per mil* (‰).

### Mapping of zooplankton δ^15^N_bulk_ and δ^15^N_Phe_ isoscapes

The latitude and longitude of the sample locations were transformed to decimal degrees, and a Z field was generated by interpolation of the δ^15^N_bulk_ values for each sampling station. The interpolation was performed using a non-statistical model, the IDW (Inverse Distance Weighting) method; entry values were assumed by the default functions in ArcMap (Version 10.7), and a search distance of five times the cell size of the output raster and a power adjustment of 2 was used. To evaluate whether δ^15^N_Phe_ values of zooplankton (fraction size of >2000 μm) reflected the bulk δ^15^N values of zooplankton (fraction size of <1000 μm), a Pearson’s linear correlation analysis was performed. The δ^15^N_bulk_ and δ^15^N_Phe_ values of zooplankton were highly correlated (r = 0.96 p<0.001; S1 Fig), although δ^15^N_Phe_ values were depleted in ^15^N by ca. 2 ‰ relative to bulk measurements. Hence, a linear model (δ^15^N_Phe_ = 0.87* δ^15^Nbulk− 2.74) was used to calculate δ^15^N_Phe_ values for stations for which CSIA-AA measurements were not performed, allowing for an δ^15^N_Phe_ based isoscape for the entire GM. Since zooplankton sampling took place in 2017, the same isotopic baseline was assumed for both YFT sampling years. The maps are in-house products constructed with the toolbox "Geostatistical Analyst" of ArcMap (Version 10.7). Shapes used to build maps were downloaded from free licensed databases [71–75].

### Bayesian stable isotope mixing model to estimate the proportional contribution of two baselines to yellowfin tuna tissues

Based on the spatial distribution of the zooplankton isotope composition, two baselines were considered: one for the northern GM and another for the central-southern GM (see Results). To assess the relative contribution of these two regions to YFT tissues, while considering variability in the isotopic composition of zooplankton collected in each region, a Bayesian stable isotope mixing model was applied.

The stable isotope mixing model is used to estimate the proportional contribution of different prey sources to consumer (YFT) tissues based on the carbon and nitrogen isotopic composition of the consumer and their prey sources, along with a TEF [76]. The trophic position for YFT was estimated as 4.2 using a Bayesian approach (see results). Their prey should occupy one trophic level lower (3.2). We assumed zooplankton are at a TP of 2, and hence their isotope ratios were used as a baseline for calculating the isotopic composition of YFT prey by correcting with the expected isotopic trophic enrichment. TEFs used in the model were the empirically derived TEFs for Pacific bluefin tuna (*Thunnus orientalis;* 62.5–75.0 cm curved fork length) held in captivity: Δ^13^C = 1.8‰ and Δ^15^N = 1.9‰ for muscle and Δ^13^C = 1.2‰ and Δ ^15^N = 1.1‰ for liver [77].

The parameters of the Bayesian mixing model were estimated through a Markov Chain Monte Carlo procedure implemented in the language Just Another Gibbs Sampler (JAGS) in Rstudio. We ran 10,000 iterations in four independent chains, with a burn-in phase of 1,000 samples to calculate the posterior distribution, and Bayesian credibility intervals were calculated. Results are reported as a proportional contribution of each baseline (%) at mode 95%. Mixing model results were estimated with the *SIMMR* package as an upgrade to the *SIAR* package [76] in Rstudio Version 1.1.463 –© 2009–2018 (R development Core Team 2008).

### Trophic position estimates

To estimate discrete TP of YFT based on δ^15^N_bulk_ values of muscle and liver (hereafter referred to as TP_bulk_) Post’s [31] equation was used (2):

$${TP}_{x}=\left(\frac{\delta^{15}N_{x}-\delta^{15}N_{Baseline}}{TEF}\right)+{TP}_{Baseline}$$ (2)

where *x* is YFT, the δ^15^N_baseline_ corresponds to the isotopic composition of primary consumers (inferred based on the isotopic mixing model), and TP_baseline_ is the TP of primary consumers. For this study, a TP_baseline_ of 2 was used based on Basedow et al. [78] for the 200–1000 μm size fraction of zooplankton in the North Atlantic. In calculating TP_bulk_ for YFT, we used TEF values of Pacific bluefin tuna (*Thunnus orientalis*) held in captivity and fed with natural diet [77] and compared the results with previous reports based on stomach content analysis (S4 Table).

Additionally, a Bayesian approach was implemented to estimate TP_Bayesian_ of YFT. This two-source mixing model incorporates two isotopic baselines and two elements (C and N) in calculating TP. δ^13^C and δ^15^N values of zooplankton from the northern (n = 28) and central-southern (n = 61) region of the GM were used as baselines (see Results). TP_Bayesian_ was estimated for YFT muscle by assuming a baseline TP of 2 and the TEFs for Pacific bluefin tuna muscle [77]. The Bayesian model was run with uninformative priors, two MCMC chains, 20,000 iterations. The *tRophic Position* package (version 0.7.7) was used in Rstudio Version 1.1.463 –© 2009–2018 (R Development Core Team 2008) [79].

To estimate the TP based on nitrogen CSIA-AA (TP_CSIA_), the equation of Chikaraishi et al. [40] was used (3):

$${TP}_{x/y}=\frac{\left(\delta^{15}N_{x}-\delta^{15}N_{y}-\beta \right)}{\left({TEF}_{x}-{TEF}_{y}\right)}+1$$ (3)

where *x* is the canonical trophic AAs (Glu), *y* is the canonical source AA (Phe), and β represents the difference between the δ^15^N values of trophic and source AAs in primary producers (β = 3.4± 0.9‰ estimate based on 17 aquatic photoautotrophs; Chikaraishi et al. [40]). This value has been used in other TP_CSIA_ estimates for YFT and Pacific bluefin tuna [80, 81]. The trophic discrimination factor (TDF) reflects the cumulative isotope discrimination of the source and trophic AA per trophic level [44, 55]. Some studies suggest that TDF values may be taxon-specific and that they may vary as a function of protein quantity and quality, as well as an organism’s TP [82, 83]. Hence, we calculated TP_CSIA_ using literature-derived TDFsand compared the results to those obtained using bulk δ^15^N values and the results of stomach content analysis in other studies.

Specifically, we used TEF estimates for muscle tissue based on bluefin tuna fed in captivity and wild captures from marine teleosts combined with diet information from the literature [52, 84]. We used TEFs for liver for the carnivorous yellowtail (*Seriola lalandi*) fed under controlled feeding conditions because that is the only study to date reporting on that tissue [85].

The propagated errors for Eqs 2 and 3 were calculated by combining the analytical reproducibility of isotopic measurements (SD of 0.2, 0.4, and 0.5‰ for bulk, Phe and Glu δ^15^N values, respectively), variation in trophic enrichment or discrimination factors and ß (0.9‰ from Chikaraishi et al. [40], following Choy et al. [86].

### Statistical analyses

Curved fork length, δ^13^C, δ^15^N, δ^15^N_Phe_, and δ^15^N_Glu_ values were tested for normality by groups (year or tissue type) using the Shapiro-Wilk test. Homoscedasticity of variance was evaluated with Bartlett’s test for groups exhibiting normality and with Levene’s test for those that failed to show normality. Differences in mean curved fork length, δ^13^C, δ^15^N, δ^15^N_Phe_, and δ^15^N_Glu_ values between either years or tissues were tested using one-way ANOVA with *post hoc* Tukey’s or Dunn’s tests for groups with a normal distribution, and with a nonparametric Kruskal-Wallis test (KWt) with a *post hoc* Mann-Whitney-U test (MWUt) when data failed to exhibit normality. The level of significance of all statistical tests was α = 0.05. Linear regression analyses of δ^13^C and δ^15^N values of muscle and liver with tuna curved fork length were done to evaluate whether the isotopic composition was correlated to YFT size. A linear regression was applied to zooplankton δ^15^N vs δ^15^N_Phe_ values to evaluate the level of correlation between these measurements and derive a model with which to estimate zooplankton δ^15^N_Phe_ values for stations for which CISA-AA were not analyzed. Analyses were performed with the *pgirmess* package in Rstudio Version 1.1.463 –© 2009–2018 (R Development Core Team 2008) and JASP Version 0.11.1.

## Results

### Bulk (δ^13^C and δ^15^N) and amino acids (δ^15^N) analyses of yellowfin tuna

**Fig 2:**
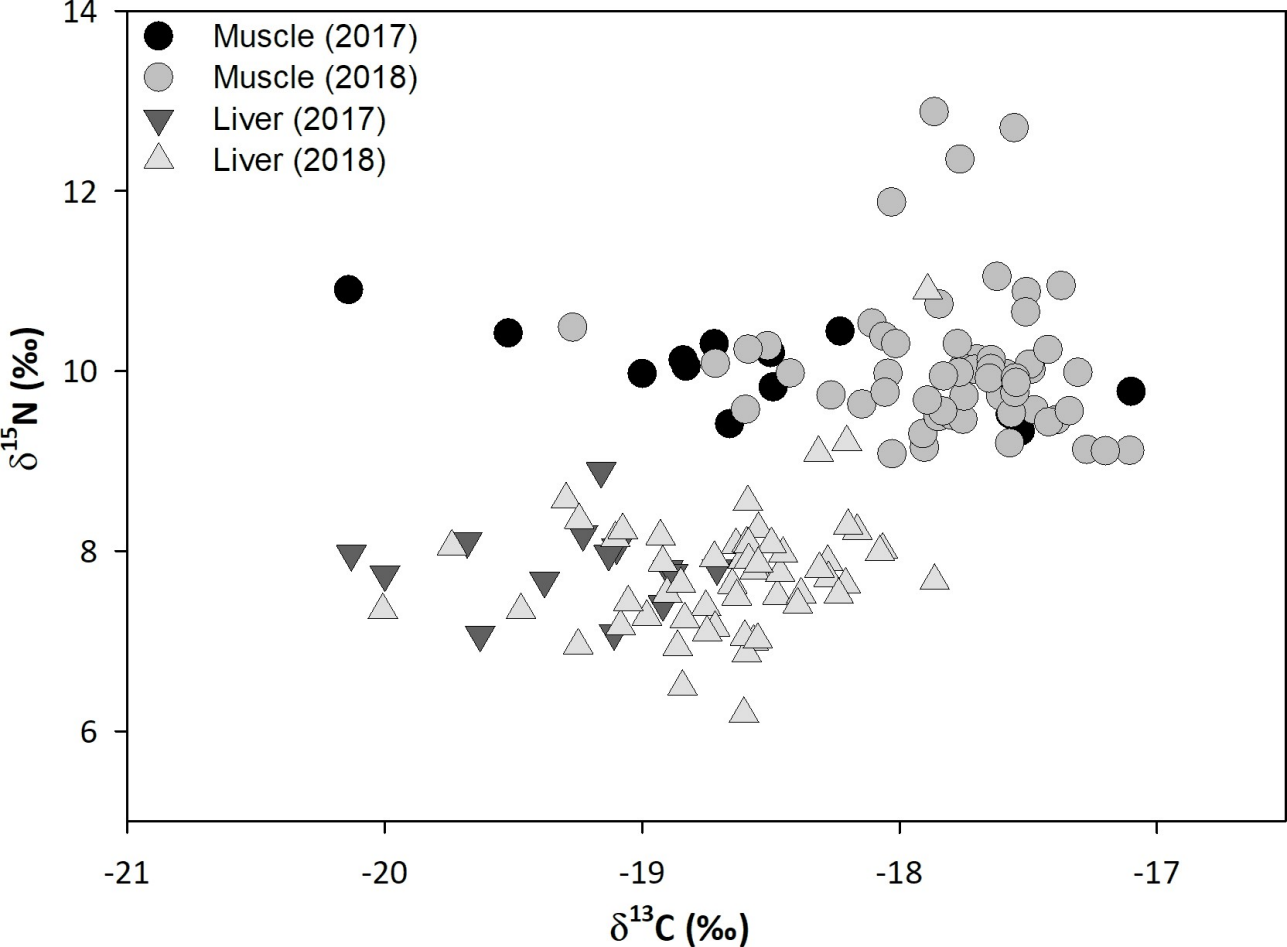
Scatterplot of bulk carbon vs. nitrogen isotope ratios. Isotopic composition of muscle (WM) and liver (LVR) tissues of 72 yellowfin tuna (*T*. *albacares*) caught in the southern (2017) and southern-central (2018) Gulf of Mexico.

There were significant differences in the mean curved fork length of YFT caught in 2017 and 2018 (one-way ANOVA, p = 0.009), although mean sizes differed by only 5 cm and the range of sizes overlapped (Table 1; S1 Table). Bulk δ^13^C values were more variable in muscle than in liver tissue within a single year, and δ^13^C values exhibited a narrower range (-20.1 to -17.1‰) than δ^15^N values (6.2 to 12.9‰; Fig 2). When comparing means of the isotopic composition measured in liver tissue, there were significant differences between years for δ^13^C values (MWUt, p<0.001) but not for δ^15^N values (one-way ANOVA, p = 0.70). For muscle tissue, there were significant differences for mean δ^13^C values (MWUt, p = 0.007), but not for δ^15^N values (one-way ANOVA, p = 0.77). A mathematical correction was refrained from δ^13^C values because C:N ratios indicated a relatively low lipid content, and corrections would have a limited (< 0.9 ‰) impact on carbon isotope ratios (see Post et al. [31]).

**Table 1:** Isotopic composition of soft tissues of YFT caught in the central and southern GM.

Year	CFL (cm)	Tissue	Bulk isotope ratios		δ^15^N of canonical source AA	δ^15^N of canonical trophic AA	C:N
			δ^13^C (‰)	δ^15^N (‰)	Phe (‰)	Glu (‰)	
2017 (*n = 14*)	134.9 ± 8.0(123 to 146)**	Muscle	-18.5 ± 0.8*** (-20.1 to -17.1)	10.0 ± 0.4 (9.3 to 10.9)	6.0 ± 1.2 (4.4 to 8.7)	26.4 ± 0.5*** (25.7 to 27.1)	3.6 ± 0.4 (3.1 to 4.4)
		Liver	-19.3 ± 0.4*** (-20.1 to -18.7)	7.8 ± 0.5 (7.1 to 8.9)	4.6 ± 0.6 (3.5 to 5.9)	20.2 ± 0.6***(18.9 to 21.8)	4.1 ± 0.3 (3.8 to 4.7)
2018 (*n = 58*)	140.5 ± 6.9(128 to 160) **	Muscle	-17.8 ± 0.4*** (-19.3 to -17.1)	10.1 ± 0.8 (9.1 to 12.9)	6.5 ± 1.4 (4.0 to 9.1)	25.3 ± 1.4*** (23.1 to 28.6)	3.3 ± 0.2 (3.1 to 3.9)
		Liver	-18.7 ± 0.4 (-20.0 to -17.9)	7.8 ± 0.7 (6.2 to 10.9)	4.1 ± 1.3 (1.7 to 6.8)	19.1 ± 0.8 (17.4 to 21.4)	4.2 ± 0.3 (3.7 to 5.2)

Number (*n*) of yellowfin tuna sampled in 2017 and 2018 and curved fork length (CFL) in centimeters. Isotopic composition (δ^13^C and δ^15^N) of bulk tissues, δ^15^N values of the canonic source and trophic amino acid (AA) phenylalanine (Phe) and glutamic acid (Glu), respectively, and carbon vs nitrogen ratio (C:N). Values are means ± one standard deviation; ranges are in parenthesis. Units are in per mil (‰). *Indicates significant differences in the same tissue between years and differences in CFL between years tested with Kruskal-Wallis and Mann-Whitney *post hoc* tests. Significance levels are indicated as: *p ≤ 0.05,** for 0.01, and *** for p ≤ 0.001.

**Fig 3:**
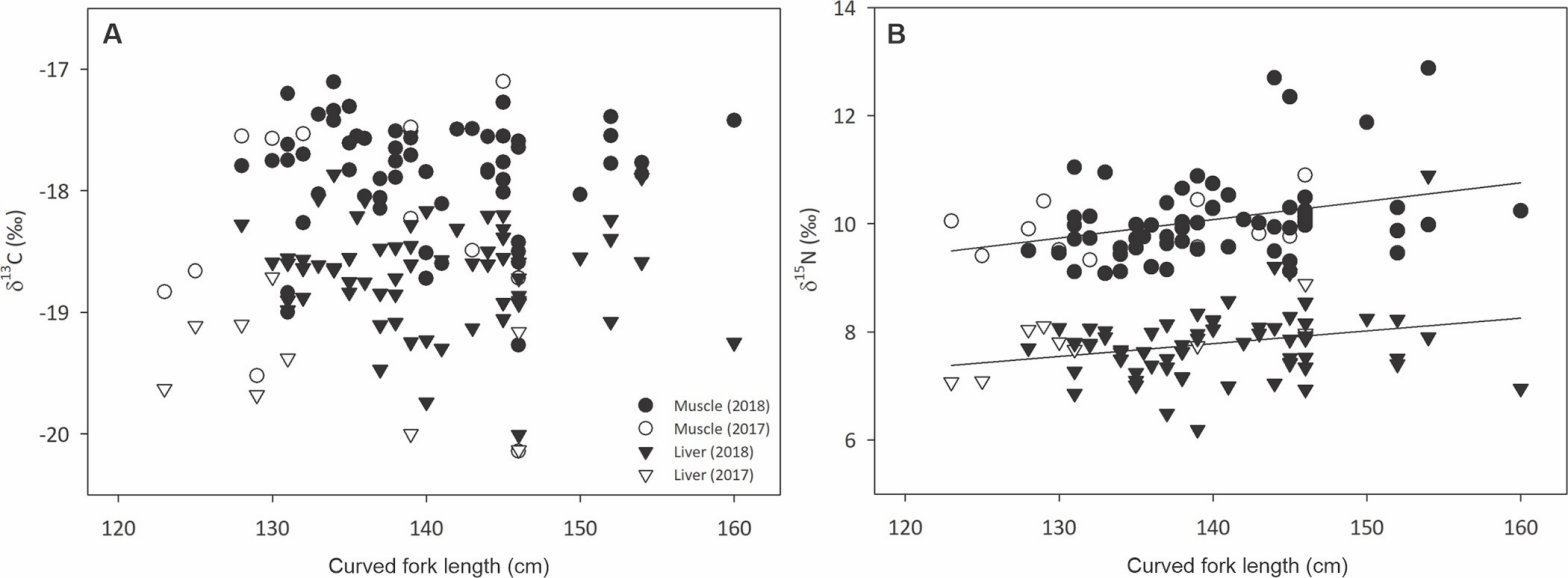
Relationship between YFT curved fork length and bulk δ^13^C and δ^15^N values of muscle and liver tissues. (A) YFT curved fork length in cm vs. bulk δ^13^C values of muscle and liver tissue. (B) δ^15^N values of muscle and liver tissue; solid lines indicate a significant linear relationship between curved fork length and δ^15^N values in muscle (r = 0.34, p = 0.003) and liver (r = 0.03, p = 0.02).

When comparing tissues, there were significant differences between both mean δ^13^C and δ^15^N values of bulk muscle and liver (MWUt, both p<0.001), and muscle tissue showed higher values for both elements. No linear relationship was found between curved fork length and δ^13^C values for either tissue (Fig 3A). In contrast, there was a weak but significant correlation (r = 0.34 and 0.03 for muscle and liver tissue, respectively) between CFL and δ^15^N values (Fig 3B). However, the best fit relationship indicates a limited (<1 ‰) difference between the smallest and largest YFT samples each year.

**Fig 4:**
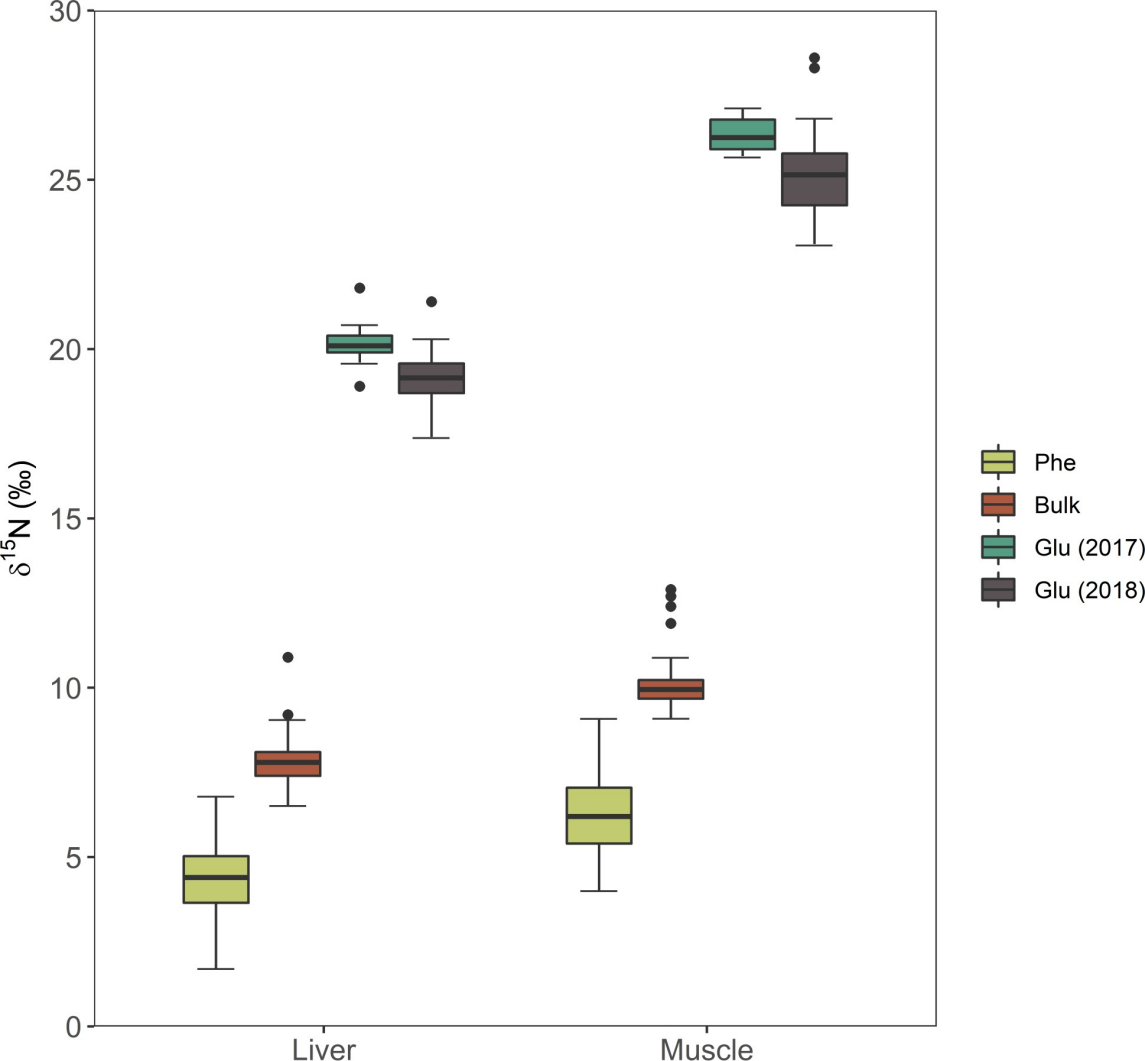
Comparison of δ^15^N of bulk tissues, phenylalanine and glutamic acid in liver and muscle tissues of YFT. Due to differences between δ^15^N values of glutamic acid, these were separated for analysis. Boxplots display the median (bold middle line), the interquartile range (box), and the minimum and maximum observations that extend to the whiskers and outlier points beyond the whiskers.

For CSIA-AA, no significant differences were found between mean δ^15^N_Phe_ values of muscle and liver tissues between years (one-way ANOVA p = 0.28 and p = 0.70, respectively; Table 1; S1 Table). However, there was a significant difference between δ^15^N_Glu_ values of muscle and liver between years (MWUt, p<0.001, and one-way ANOVA, p<0.001, respectively; Fig 4). Both δ^15^N_Phe_ and δ^15^N_Glu_ values were higher in muscle than in liver tissue. Isotopic datasets that did not exhibit statistical differences between years were pooled for subsequent analyses.

### Isoscapes and region-specific baseline values

**Fig 5:**
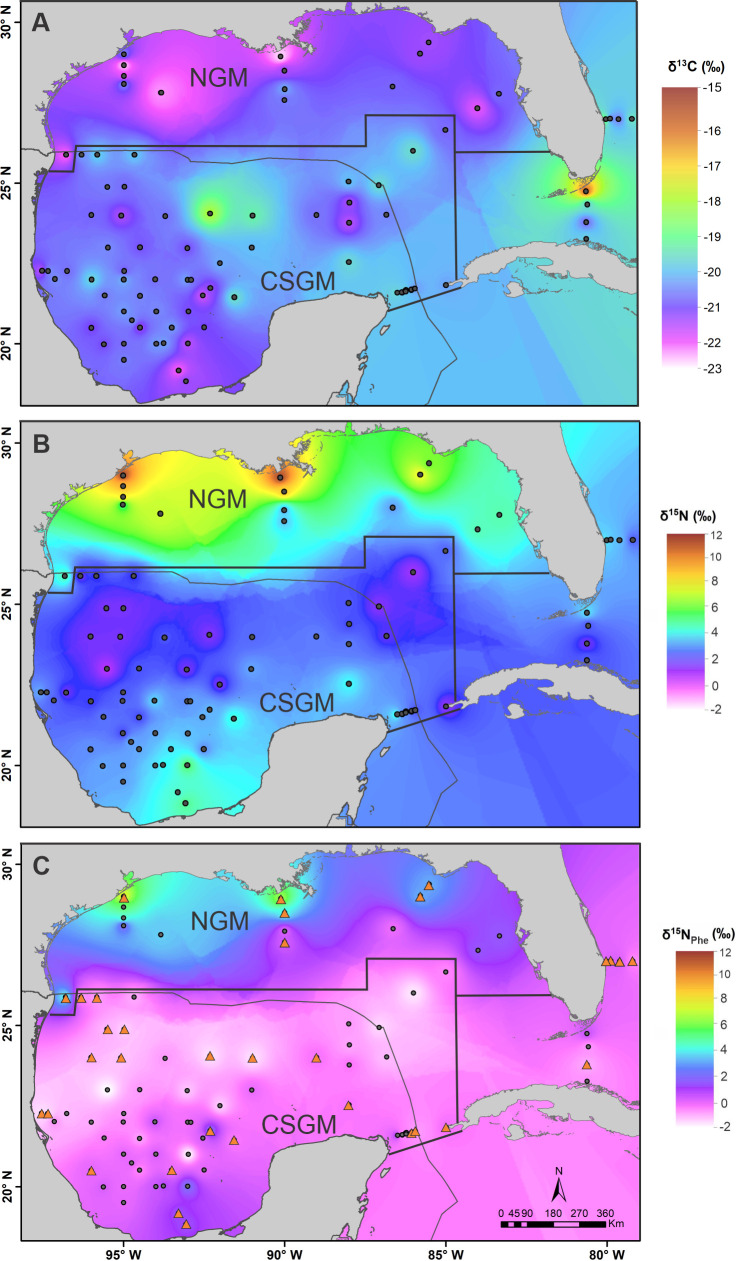
Gulf of Mexico isoscapes. Gulf of Mexico (GM) zooplankton-based (A) δ^13^C and (B) δ^15^N isoscape generated from 335–1000 μm zooplankton using an IDW interpolation. The solid black polygon delimits the stations encompassed in each of the two baseline regions: the northern (NGM) and central-southern gulf (CSGM). (C) δ^15^N_Phe_ isoscape using an IDW interpolation, based on direct measurements of Phe in zooplankton size > 2000 μm (orange triangles) and completed with data from a linear regression model relating δ^15^N_bulk_ and δ^15^N_Phe_ (dark dots). The grey line delineates the Mexican Exclusive Economic Zone. Layers of country limits used to build maps were downloaded from free licensed data bases available at: https://www.inegi.org.mx/app/mapas/, http://www.diva-gis.org/gdata.

The mean (±SD) bulk δ^13^C values for zooplankton collected throughout the GM in 2017 was -20.3 ± 1.1‰, ranging from -22.7 to -14.8‰ (S2 Table). Stations sampled in the northwestern shelf had isotope ratios that were relatively depleted in ^13^C compared to the central and southern GM (-22.7 to -21.0‰; Fig 5A). The mean zooplankton bulk δ^15^N value was 3.5 ± 2.1‰, with a very broad range of isotope ratios (0.9 to 11.6‰). The δ^15^N isoscape showed a strong latitudinal gradient from the northern to central-southern GM (Fig 5B). Higher δ^15^N values were observed in the coastal waters and shelf off Texas, Louisiana, and Mississippi, and lower values were measured in the deep water region of the central gulf. The eastern portion of the Bay of Campeche had more enriched δ^15^N values than the central gulf (4.5 to 6.8‰), but the isotopic composition was not as enriched in ^15^N as that of those sampled from the coast and shelf of the northern gulf.

Based on the marked north-to-south spatial gradient in baseline isotope ratios, the GM was divided into two regions: the northern GM (n = 28) and the central-southern GM (n = 61). Mean baseline values for each region were calculated. For the northern GM, mean δ^13^C and δ^15^N values for zooplankton were -21.3 ± 0.9‰ (range -22.7 to -19.9‰) and 6.8 ± 2.6‰ (3.1 to 11.6‰), respectively, and for the central-southern GM -20.1 ± 0.8‰ (-21.9 to -17.3‰) and 2.7 ± 0.9‰ (0.9 to 5.5‰), respectively (S2 Table).

Similar to the patterns observed for the δ^15^N isoscape, the δ^15^N_Phe_ isoscape exhibited a strong gradient from north to south (Fig 5C). Northern GM δ^15^N_Phe_ values decreased latitudinally from 8.5‰ near the coast of Louisiana and Texas to 3.2‰ southward (S6 Table). In the deep water region of the GM, δ^15^N_Phe_ values were between -2.0 and 0‰, while in the southeastern Campeche Bay, δ^15^N_Phe_ values were somewhat higher (ca. 1.9‰).

### Yellowfin tuna foraging habitat within the Gulf of Mexico

**Fig 6:**
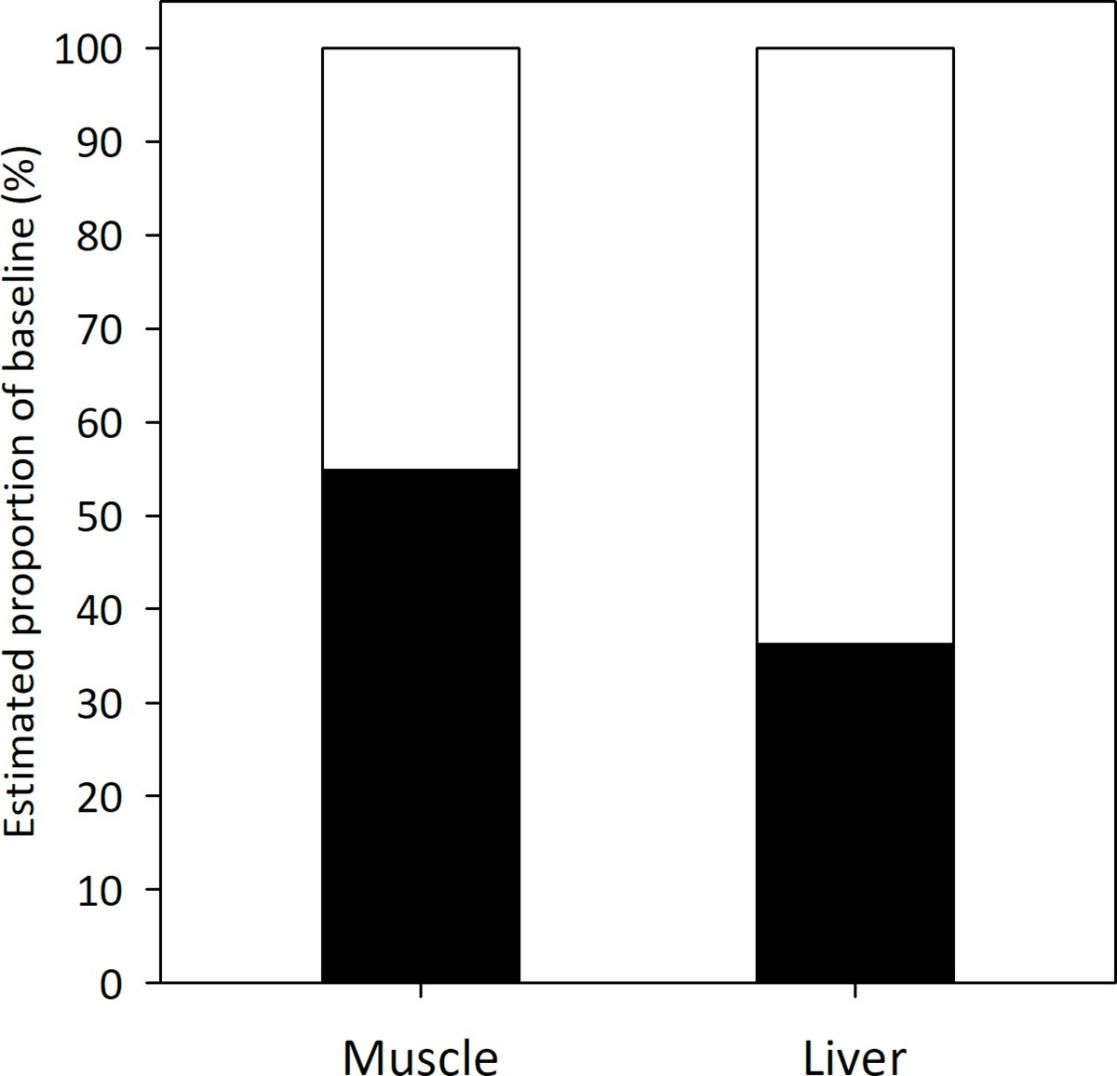
Relative contribution of two regional baselines to yellowfin tuna tissues. Isotopic baseline contributions (%) of the northern (black) and central-southern (white) Gulf of Mexico to yellowfin tuna caught in the southern and central region of the GM, muscle and liver tissues based on a two-source Bayesian mixing model.

Bayesian mixing models were used to estimate the relative contribution of the TEF-corrected baselines of the northern and central-southern GM to YFT tissues. Results for muscle tissue (estimated isotope integration time of ~334 days [77]) indicate that the contribution of the northern GM to YFT nitrogen values was 54.9% [48.7–62.1%], compared to 45.1% [37.9–51.3%] for the central-southern region. On the other hand, results for liver tissue (estimated isotope integration time of ~172 days [77]) suggest that more recently, YFT fed to a greater extent in the central-southern GM; the contribution of the southern baseline was higher, 63.7% [55.0–71.7%] compared with that for the northern GM of 36.3% [28.3–45.0%] (Fig 6).

**Fig 7:**
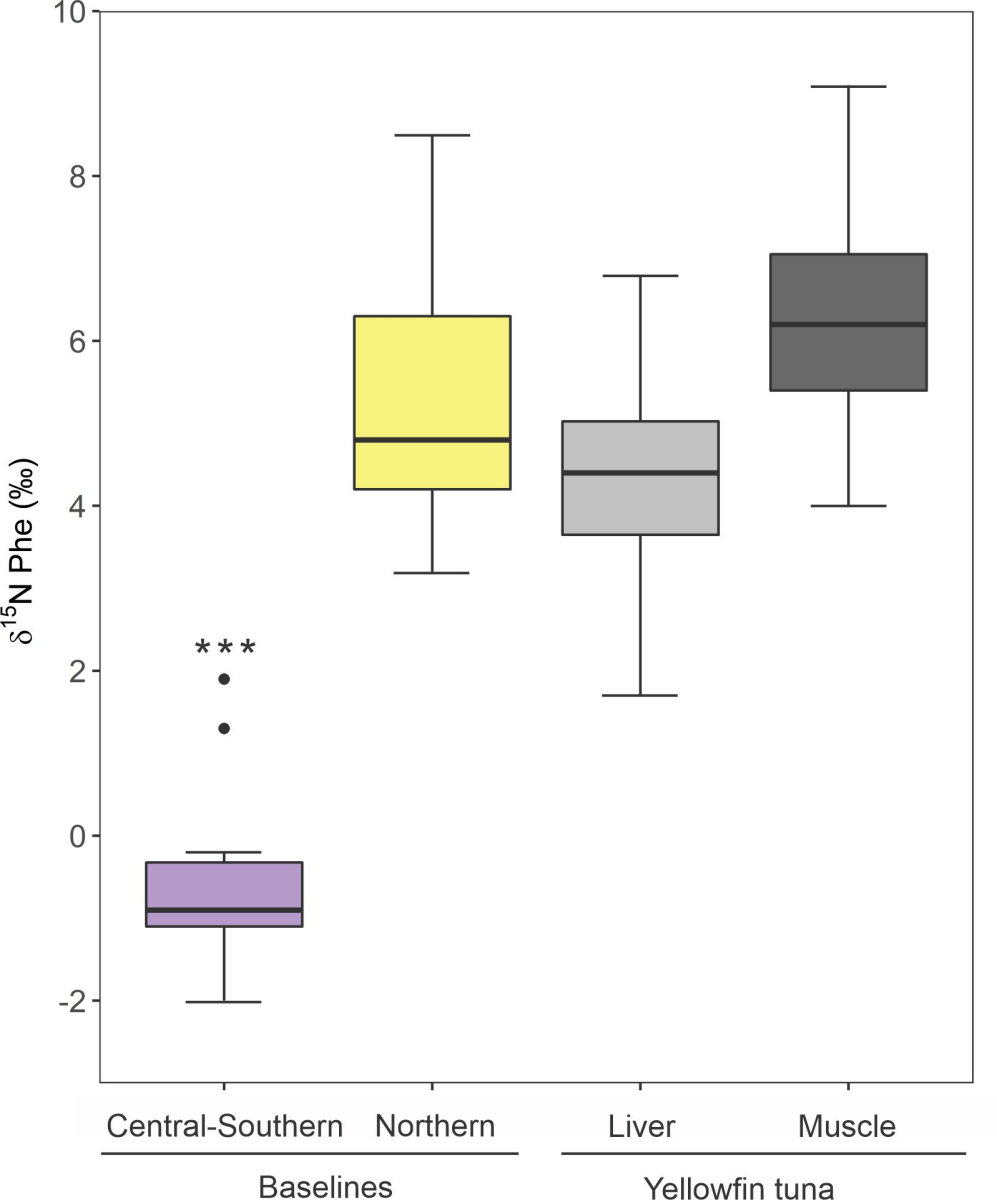
Comparison of δ^15^N_Phe_ values of zooplankton and yellowfin tuna liver and muscle tissues. Purple and yellow box plots represent the central-southern (n = 14) and northern (n = 6) GM baselines, respectively. Light grey and dark grey are δ^15^N_Phe_ values of liver and muscle of yellowfin tuna, respectively (n = 36). Boxplots display the median (bold middle line), the interquartile range (box), minimum and maximum observations that extend to the whiskers and outlier points beyond the whiskers. Stars show statistical differences in the central-southern GM baseline value with the northern GM baseline and yellowfin tuna tissues based on post-hoc Tukey’s tests.

There were significant differences between mean YFT muscle and liver tissues and central-southern GM δ^15^N_Phe_ values (Tukey test, both p<0.001). No statistical differences were found between mean zooplankton δ^15^N_Phe_ values of the northern GM baseline and muscle and liver tissues (Tukey test, p = 0.25 and p = 0.31, respectively), suggesting that the isotopic composition of the source AA in YFT tissues reflects the isotopic baseline of the northern GM (Fig 7).

### Yellowfin tuna trophic position estimates

Because there were no significant differences in the δ^15^N_bulk_ values of muscle and liver tissues between years (one-way ANOVA, p = 0.77 and p = 0.70, respectively), data for each tissue were pooled for estimating TP_bulk_. Estimates of TP_bulk_ based on δ^15^N values of YFT muscle and liver and the northern GM zooplankton as the sole isotopic baseline were 4.1 ± 0.4 and 5.7 ± 0.7, respectively (Table 2, S3 Table). Estimates of TP_bulk_ based on the central-southern GM region baseline were markedly higher, with values of 5.8 ± 0.4 for muscle and 8.6 ± 0.7 for liver tissue, which is well above the estimates previously reported for this species. TP_bulk_ estimates based on liver δ^15^N values were higher and more variable than those based on muscle (S3 Table). Uncertainty in TP based on bulk SIA analysis of muscle tissue and propagated error calculations were ±1.8 and 0.9 when using the northern and southern isotopic baseline, respectively. For liver tissue, the uncertainty was substantially higher (±3.8 and 3.1, respectively). For CSIA, it was ±1.0 and 0.7 TP for muscle tissue estimated using tuna and marine teleost TDFs, respectively, and ±2.5 for liver tisse.

**Table 2:** Mean trophic position (TP) estimates for yellowfin tuna caught in the central and southern Gulf of Mexico (GM) based on muscle tissue.

TP estimation method	Source	Organism and approach for estimations of TEFs or TDFs	TEF (bulk) or TDF (CSIA-AA) (‰) (Mean ± SD)	Calculated TP	
				Northern GM	Central-southern GM
Bulk δ^15^N values	Madigan et al. [77]	Pacific bluefin tuna (*Thunnus orientalis*) held in captivity and fed with natural diet	1.9 ± 0.4	**4.1 ± 0.4**	**5.8 ± 0.4**
Bayesian (Bulk δ^13^C and δ^15^N)			N = 1.9 ± 0.4 C = 1.1 ± 0.6	**4.2 [4.0–4.4]**	
CSIA-AA	Bradley et al. [52]	Pacific bluefin tuna (*Thunnus orientalis*) held in captivity and fed with natural diet	6.3 ± 0.4	**3.5 ± 0.2**	
	Bradley et al. [84]	Wild captures from marine teleosts combined with diet information from the literature	5.7 ± 0.3	**3.8 ± 0.3**	

TP calculated from three approaches: (1) using YFT δ^15^N_bulk_ values and the northern GM and central-southern GM regional isotopic baselines with mean δ^15^N values of 6.0 ± 3.1‰ and 2.8 ± 1.0‰, respectively; (2) a Bayesian analysis that integrates YFT bulk δ^15^N and δ^13^C values and two regional baselines and YFT tissues; and (3) δ^15^N_Phe_ of YFT and zooplankton. TEFs for TP_bulk_ estimates and trophic discrimination factors (TDF = TEF_Glu-_TEF_Phe_) for TP_CSIA_ estimates derived from the literature.

In contrast, TP calculated with the Bayesian approach and considering both isotopic baselines as source contributions was 4.2 [4.0–4.4]. This value and the credibility interval is highly consistent with TP estimates for YFT derived from the literature that are based on SIA_bulk_ (4.1) and stomach content analysis (4.0, S4 Table). There was a significant but low correlation between curved fork length and all TP_bulk_ estimates (for all TEFs applied and both tissues) with an overall r<0.34 (Pearson’s correlation p< 0.006 for all cases, S5 Table).

TP_CSIA_ estimates based on YFT muscle values had a mean of 3.8 ± 0.3. Applying the only available TDF for fish liver tissues yielded a mean TP of 3.9 ± 0.2 (S3 Table). Overall, TP_CSIA_ estimates (coefficient of variation, 7.8%) were less variable than those of TP_bulk_ (CV = 28.1%). There was no correlation between curved fork length and TP_CSIA_ (Pearson’s correlation p> 0.31 for all correlations, S5 Table).

## Discussion

The ẟ^15^N_bulk_ and ẟ^15^N _Phe_ values of zooplankton measured throughout the GM showed a strong geographical gradient that allowed for the separation of the basin into two regions. Specifically, the ẟ^15^N based isoscape exhibited a clear latitudinal gradient, with higher values in the north (3.1 to 11.6‰ and 3.2 to 8.5‰ for bulk and Phe, respectively) and lower values in the central-southern region (0.9 to 5.5‰ and -2.0 to 1.9 ‰ for bulk and Phe, respectively). The regional differences in nitrogen isotopic composition of secondary consumers (zooplankton) indicate that in order to make reliable inferences about the feeding history and TP of YFT within the GM, the ẟ^15^N variability in these biogeochemical regions must be considered.

The ẟ^15^N values of zooplankton samples collected near the Mississippi River plume and on the Texas and Louisiana shelves (3.3 to 8.9‰) were similar to those reported (2.6 to 7.8‰) by Dorado et al. [61]. High ẟ^15^N values in the northern gulf have been associated with high inputs of freshwater discharge transporting high concentrations of dissolved nitrate from anthropogenic activities such as intensive livestock production in the central United States, inputs of treated wastewater and N derived from manure [87–90]. In addition, large inputs of nutrients to coastal and shelf waters can also result in an enhancement of primary and often secondary production, which aggravates hypoxia and intensifies denitrification [87, 91, 92]. This process has a large fractionation of ~25‰ that leads to a marked enrichment of ^15^N in the isotopic composition of the inorganic nitrogen pool [47]. The high ẟ^15^N values observed in the northern GM have been shown to reflect intense denitrification, leading to nitrate enriched in ^15^N [93, 94]. Hence, high ẟ^15^N values of zooplankton in the shelf and coastal areas of the northern GM lead to a region with a distinct isotopic baseline.

In contrast to the northern GM, in the central oceanic region, very low zooplankton ẟ^15^N values of 0 to 2‰ were measured. Low ẟ^15^N values at the base of the food web in the GM have been linked to oligotrophic conditions, particularly anticyclonic eddies that are characterized by a deepening of the thermocline that limits the subsurface transport of new nitrogen (nitrate) to the surface [61, 95, 96]. *Trichodesmium*, a diazotrophic nitrogen-fixing bacteria, is abundant in the surface waters of anticyclonic eddies and in the deep water region (depths > 1000 m) of the GM [95, 97]. Diazotrophic bacteria are responsible for nitrogen inputs to the surface via atmospheric N_2_ fixation, a process that leads to minimal isotope discrimination. Hence, fixed nitrogen has an isotopic composition of ~0‰, similar to atmospheric nitrogen [47]. These relatively low values are reflected in the zooplankton collected in the central and southern GM.

Zooplankton from a few stations within the Bay of Campeche exhibited ẟ^15^N values enriched in ^15^N relative to the central gulf (2.9 to 5.5‰ and -0.7 to 1.3‰ for bulk and Phe, respectively), although they were not as high as those observed in the northern GM. These regionally higher values were observed only in the southwestern reaches of the bay, where the Grijalva-Usumacinta river system discharges onto the continental shelf and cross-shelf transport has been documented in late summer and early fall [98]. In this region, upwelled water that is transported across the Yucatan shelf can also be found [64, 65]. Upwelled water has a high nitrate concentration, with an isotopic composition presumably similar to that reported for eastern Atlantic subsurface waters (~4.7‰ [99]). Nevertheless, the ẟ^15^N values of zooplankton from the southern bay of Campeche are not as enriched as those found in the northern GM regardless of the source of nitrogen enriched in ^15^N, also these are limited in spatial distribution. Hence, they are unlikely to contribute substantially to the isotopic composition of YFT.

### Yellowfin tuna foraging habitat in the Gulf of Mexico

One key aspect to making inferences about the origin and timing of previous feeding habitats of animals that move between isotopically distinct regions is to have adequate estimates of the isotopic integration time (a function of isotopic turnover rate) of the tissue of interest [42].

Based on the ẟ^15^N values of muscle tissue, the Bayesian mixing model indicated that the northern GM baseline contributed a higher proportion (54.9%) than the southern GM (45.1%) to YFT. Assuming that the isotopic composition of the baselines does not change substantially over the year, this implies that YFT fed to a greater extent in the northern gulf. On the other hand, the more recent feeding habitat, as reflected by liver tissue with a shorter time to equilibrium, exhibited a higher contribution of the central-southern GM baseline (63.7%) compared to the northern GM (36.3%). Hence, YFT had fed more recently in the central-southern GM in which they were caught.

Given the strong correlation between zooplankton δ^15^N_bulk_ and δ^15^N_Phe_, the range of δ^15^N_Phe_ values of both muscle and liver tissue also indicate that the northern gulf is an important feeding habitat. As a source AA with little or no isotope discrimination [59], δ^15^N_Phe_ values of YFT tissues should reflect those of primary producers and secondary consumers. The δ^15^N_Phe_ of YFT muscle (6.2 ± 1.3‰) and liver (4.1 ± 1.3‰) were not significantly different from the northern GM isotopic baseline values, consistent with feeding in the region. However, mean liver δ^15^N_Phe_ values were lower (2.2‰ lower) than those of muscle tissue, which implies more recent feeding in the central-southern GM, as was found with the bulk data. Therefore, both the bulk SIA and CSIA-AA suggest that the main foraging ground of the YFT within the GM is the northern region, although feeding occurs in the central and southern gulf as well. Unfortunately, inferences about the time-integrated could not be assessed with the source AA, since there are no published isotope turnover rates for δ^15^N_Phe_ of large pelagic species such as YFT.

The findings of this study suggest that the northern GM is an important foraging region for YFT, as has been documented for other highly migratory pelagic species that use the northern GM as spawning and feeding ground [100, 101]. In the case of YFT, tagging studies in this region show limited movements (<150 km) and a high degree of regional residency [17, 19]. In addition, the presence of nearly 4,000 oil rigs in the northern gulf may serve as areas of aggregation and provide foraging opportunities that may contribute to the residency of YFT in the northern gulf [17, 102]. The northern GM also provides optimal conditions for the successful growth and survival of YFT larvae, such as high values of surface chlorophyll-a (i.e., high productivity) and intermediate salinities. These conditions are observed near the Mississippi River plume, where freshwater and oceanic waters mix, and where a high abundance of YFT tuna larvae has been found [11, 12].

Within the Mexican Exclusive Economic Zone, YFT supports an important fishery that operates year-round [4]. This may be due to the high productivity of the Bay of Campeche [62, 98]. During winter, the Mexican fleet from the southern GM moves northwards toward U.S. waters, presumably "following" the abundance of YFT (Zurisaday Ramírez, Personal communication with the Mexican fishermen). Abad-Uribarren et al. [5] report an increase in catch per unit effort in the southern GM during summer months, whereas a second increase occurs during November in the northern-central GM, which supports the anecdotal information provided by Mexican fishermen. Rooker et al. [19] documented the southward movement in the fall of some of the YFT caught and tagged in the northern GM, as well as the northward migration from the south of other individuals. Together, results suggest a certain degree of seasonality in feeding.

However, there are no studies focusing on YFT foraging migrations relative to the distribution of their potential prey at a basin-wide scale. Elucidating the role of prey distribution on migration patterns can be challenging since YFT is a generalist predator that feeds on a wide array of prey [103–105]. Nevertheless, more research is necessary to understand what drives the movement patterns within the GM, especially in the central and southern gulf. Electronic tagging in the southern GM would elucidate movement patterns and habitat use, as has been achieved in the north [19].

In the temporal context of liver tissue (~6 months), our results indicate that YFT had been foraging mainly in central-southern GM. Given that the tuna in this study were caught and sampled in July and August, the feeding period reflected by liver tissue partially overlaps with the species spawning season within the gulf (May through August). The southern GM may thus serve as an important spawning and foraging ground for YFT, although larval surveys are scarce in this region. Evaluation of the spatial and temporal distribution of the larvae would help determine whether the southern GM should be reconsidered as an important spawning ground. It is important to note that our interpretation assumes a closed population of tuna within the gulf (all tuna sampled were GM residents). However, there is overlap in the bulk isotopic baselines between oceanic regions that could confound our interpretation. Similar δ^15^N values (~3 to 8‰) to those of the northern GM have been recorded in one of the YFT Eastern Atlantic spawning grounds, the Gulf of Guinea [106]. The possibility of YFT migration to the GM from the Eastern Atlantic cannot be discarded and should be evaluated using additional intrinsic tracers such as otolith microchemistry [14].

### Yellowfin tuna trophic position in the Gulf of Mexico

In fish, length is positively related to TP and gape size (i.e., larger fish can feed on larger prey [107]). The low correlation and high variability in YFT δ^15^N values of both tissues as a function of size are in agreement with previous reports for this species in other regions of its distribution [108–111]. For example, Ménard et al. [104] found that the size distribution of prey in YFT stomachs was very asymmetrical, and that large YFT continue to feed on small prey during their life. This could be due to the higher availability of smaller prey relative to larger prey in the oligotrophic surface layer, where YFT spend more time [17]. In addition, the size range of the fish sampled in this study was small (123–160 cm curved fork length), which should limit the relationship between TP and size. The lack of a strong relationship between curved fork length and δ^15^N values of muscle and liver tissues allowed us to disregard size when estimating TP.

Differences in the isotopic baselines contribute to variation in bulk δ^15^N values of YFT that inhabit a particular region, and thus impact TP estimates [44, 53, 80, 110, this study]. TP_bulk_ estimates using δ^15^N values of muscle tissue and the mean isotopic composition of northern GM as baseline yielded a TP of 4.1, which is similar to that reported for other regions throughout the species distribution (range 3.3 to 4.7; S4 Table). Estimates were likely reasonable because, for muscle tissue, the dominant source of N was estimated to be the northern GM. Hence, a single baseline yielded an adequate first approximation to TP. Nevertheless, the TP estimate based on the results of the Bayesian model yielded a more robust estimate. This approach performs a simple mixing model that allows for differentiation between two sources of N, and considers the heterogeneity of the two baselines [79]. The result was a mean TP of 4.2 [4.0–4.4], which is highly consistent with the global TP range of 3.3 to 4.7 from the literature. Hence, this approach proved to provide the most realistic approximation. As has been previously noted for other regions throughout the broad distribution of YFT, bulk δ^15^N values of muscle tissue provide robust TP estimates when the isotopic baseline is well characterized [i.e.,24, 29, 110].

In contrast, when TP_bulk_ is calculated based on muscle tissue and the mean central-southern GM baseline values, TP estimates was higher (TP 5.8) than those calculated with the northern baseline. Likewise, when the central-southern GM baseline and δ^15^N values of liver tissue are used, TP_bulk_ was unreasonably high and well above TP estimates for YFT reported for other regions of its distribution (TP 8.6 vs. global range from 3.3 to 4.7). Liver TEF values are lower and more variable than those of muscle, which contributed to the higher estimates of TPs. The lower TEF in the liver may be due to differences in its AAs composition compared with muscle, as well as its higher metabolic rate [111]. These unreasonably high TP calculated for liver tissue suggest that δ^15^N values of this tissue may not be a good predictor of TP.

TP_CSIA_ of the source and trophic AA yielded a TP range of 3.5 to 3.9. These TP are similar to those reported for YFT in other regions of its distribution based on stomach content analysis (range 3.7 to 4.3; S4 Table) but were slightly lower than those estimated with δ^15^N_bulk_. TP_CSIA_ appears to underestimate the TP of taxa at or near the top of the food web, Bradley et al. [84] suggested that the enrichment between trophic and source AAs is lower in higher TP consumers compared with those that feed at lower trophic levels, which may be due to a higher protein consumption of carnivorous diets [82, 112, 113]). Although ideally species and tissue specific-TEFs should be used to estimate TP, these empirical estimates are challenging to obtain for large predators such as YFT, and we used the best available estimates.

TP estimates also varied among individuals, which likely variability reflects a varied diet on prey of different trophic levels, rather than the feeding habits of a strict tertiary carnivore (commonly represented by the discrete trophic level of 4, Madigan et al., [114]). YFT feeds on a wide variety and sizes of prey, from small low TP pelagic crustaceans and gelatinous organisms, as well as on higher TP organisms, such as fishes and cephalopods, that explain the inshore-offshore TP pattern observed in other oceans [109, 111]. YFT can also feed on mesopelagic prey by occasionally expanding their vertical feeding range, although to a lesser extent than bigeye or bluefin tuna [24, 115]. Although stomach content analysis was not performed in this study, given the strong similarity with TP between YFT populations in other regions of its distribution, similar prey items are expected in the YFT diet within the GM.

Seasonal variation in baseline isotope ratios will impact TP estimates [116]. However, sampling of zooplankton in the central and southern Gulf of Mexico conducted during 5 cruises spanning 2010 to 2016 during different times of year indicate limited variation in mean δ^15^N values (differences in mean values ≤ 0.5 ‰; S. Z. Herzka unpublished data). Hence, temporal variation in zooplankton values in the Gulf of Mexico will likely have less of an impact on TP estimates than spatial variation.

Additionally, Some research has documented shifts in YFT feeding patterns over decadal time scales, which may be due to changes in food web structure due to overfishing and/or climate change [27]. Sibert et al. [117] analyzed the TP of exploited tunas in the Pacific Ocean and found that TP did not show an overall temporal decline over the last 60 years. In the northwestern Atlantic, tuna diets and TP have remained stable for the last 50 years [111]. However, a different pattern was observed for YFT in the eastern tropical Pacific during the early 1990s to 2000s, where a diet shift from larger epipelagic fish to a smaller mesopelagic species was documented over decadal time scales [27]. Although past estimates of TP are unavailable for YFT in the GM, the results derived from this study provides a useful baseline for future studies on their trophic ecology.

## Supporting information

S1 Fig Linear correlation model of bulk δ^15^N versus δ^15^N of phenylalanine (Phe). The broken line is a reference 1:1 line.
(DOCX)

S1 Table Summary information and isotopic composition of YFT caught in the southern Gulf of Mexico. Individual number (ID), collection year, and curved fork length (CFL) in centimeters. Isotopic composition of both white muscle and liver tissues: δ^13^C, δ^15^N, C:N ratio, δ^15^N of the canonical source and trophic amino acids (AA) phenylalanine (Phe) and glutamic acid (Glu), respectively. Units are in per mil (‰).
(DOCX)

S2 Table Summary of zooplankton sampling cruises. Cruise name, stations, coordinates in decimal degrees, and δ^13^C and δ^15^N values of zooplankton (fraction size 335–1000 μm). *Stations used as northern Gulf of Mexico (GM), ^†^Stations used as central-southern GM.
(DOCX)

S3 Table Mean trophic position based on liver tissue. Estimates for yellowfin tuna caught in the central and southern Gulf of Mexico (GM).
(DOCX)

S4 Table Mean trophic position (TP) estimates for yellowfin tuna sampled throughout its distribution. Estimates based on stomach content analysis (SCA), bulk δ^15^N analysis, and CSIA-AA δ^15^N of muscle tissue. The standard deviation is presented when reported by the authors or when it could be calculated from the raw data.
(DOCX)

S5 Table Pearson’s correlation between curved fork length (CFL) and each trophic position (TP). Estimates using five literature derived TEFs using bulk δ^15^N analyses (SIA) and two baselines and TP derived from δ^15^N values of Glu and Phe (CSIA). *indicates a significant relationship between curved fork length (CFL) and trophic position (TP).
(DOCX)

S6 Table Zooplankton isotopic composition by regions of the Gulf of Mexico (GM). Stations classified as northern GM and central-southern GM, and outside the GM (OGM). δ^15^N values of zooplankton (fraction size >2000 μm) of the canonical source amino acid (Phe).
(DOCX)
