# Supplementary material for: Yellowfin tuna (Thunnus albacares) foraging habitat and trophic position in the Gulf of Mexico based on intrinsic isotope tracers
Source: PLoS One. 2021 Feb 24;16(2):e0246082. doi: 10.1371/journal.pone.0246082 (PMC7904200; doi:10.1371/journal.pone.0246082)
Supplement: S1 Fig — The broken line is a reference 1:1 line. (DOCX) [file pone.0246082.s001.docx]

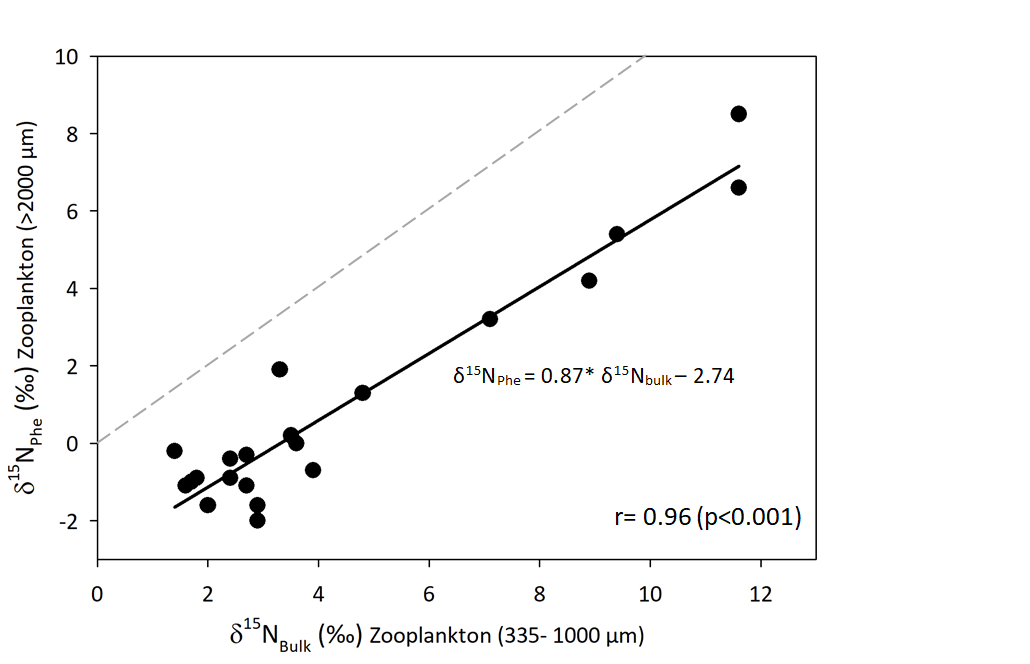


**S1 Fig. Linear correlation model of bulk δ^15^N versus δ^15^N of phenylalanine (Phe).** Linear correlation model of bulk δ^15^N values versus δ^15^N values of phenylalanine (Phe). The broken line is a reference 1:1 line.
