# Supplementary material for: Yellowfin tuna (Thunnus albacares) foraging habitat and trophic position in the Gulf of Mexico based on intrinsic isotope tracers
Source: PLoS One. 2021 Feb 24;16(2):e0246082. doi: 10.1371/journal.pone.0246082 (PMC7904200; doi:10.1371/journal.pone.0246082)
Supplement: S1 Table — Individual number (ID), collection year, and curved fork length (CFL) in centimeters. Isotopic composition of both white muscle and liver tissues: δ13C, δ15N, C:N ratio, δ15N of the canonical source and trophic amino acids (AA) phenylalanine (Phe) and glutamic acid (Glu), respectively. Units are in per mil (‰). (DOCX) [file pone.0246082.s002.docx]

**S1 Table**. **Summary information and isotopic composition of YFT caught in the southern Gulf of Mexico.** Individual ID, collection year, curved fork length in centimeters, δ^13^C, δ^15^N, δ^15^N values of the canonic source and trophic amino acids (AA) phenylalanine (Phe) and glutamic acid (Glu), respectively values are in per mil (‰), and C:N for white muscle and liver of yellowfin tuna caught in southern Gulf of Mexico.

| **ID** | **Collection Year** | **CFL** | **Muscle** | | | | | **Liver** | | | | |
| --- | --- | --- | --- | --- | --- | --- | --- | --- | --- | --- | --- | --- |
|  |  |  | **δ^13^C** | **δ ^15^N** | **C:N** | **δ ^15^N_Phe_** | **δ ^15^N_Glu_** | **δ^13^C** | **δ ^15^N** | **C:N** | **δ ^15^N_Phe_** | **δ ^15^N_Glu_** |
| 1 | 2017 | 123 | -18.8 | 10.1 | 3.77 | 6.2 | 25.9 | -19.6 | 7.1 | 4.22 | 5.3 | 18.9 |
| 2 | 2017 | 125 | -18.7 | 9.4 | 3.65 | 5.4 | 25.9 | -19.1 | 7.1 | 3.81 | 4.0 | 19.8 |
| 3 | 2017 | 128 | -17.6 | 9.9 | 3.19 | 5.0 | 26.8 | -19.1 | 8.0 | 3.98 | 4.5 | 19.6 |
| 4 | 2017 | 129 | -19.5 | 10.4 | 4.21 | 6.7 | 27.1 | -19.7 | 8.1 | 4.51 | 5.9 | 20.4 |
| 5 | 2017 | 130 | -17.6 | 9.5 | 3.20 | 7.0 | 25.7 | -18.7 | 7.8 | 3.82 | 5.0 | 20.1 |
| 6 | 2017 | 131 | -19.0 | 10.0 | 4.02 | 5.8 | 26.2 | -18.9 | 7.8 | 3.94 | 4.4 | 19.9 |
| 7 | 2017 | 131 | -18.8 | 10.1 | 3.75 | 4.4 | 26.1 | -19.4 | 7.7 | 4.12 | 4.7 | 20.0 |
| 8 | 2017 | 132 | -17.5 | 9.3 | 3.23 | 6.6 | 25.7 | -18.9 | 7.8 | 4.01 | 4.4 | 19.9 |
| 9 | 2017 | 139 | -18.2 | 10.4 | 3.37 | 4.4 | 26.3 | -20.0 | 7.7 | 4.54 | 3.5 | 20.6 |
| 10 | 2017 | 140 | -18.7 | 10.3 | 3.70 | 6.7 | 27.0 | -19.2 | 8.2 | 4.09 | 4.3 | 20.1 |
| 11 | 2017 | 143 | -18.5 | 9.8 | 3.64 | 5.5 | 26.6 | -19.1 | 8.0 | 4.07 | 4.5 | 20.4 |
| 12 | 2017 | 145 | -17.1 | 9.8 | 3.12 | 8.7 | 25.9 | -18.9 | 7.4 | 3.82 | 5.2 | 20.7 |
| 13 | 2017 | 146 | -20.1 | 10.9 | 4.44 | 6.2 | 27.0 | -19.2 | 8.9 | 4.26 | 5.1 | 21.8 |
| 14 | 2017 | 146 | -18.5 | 10.2 | 3.64 | 5.4 | 26.7 | -20.1 | 8.0 | 4.73 | 4.2 | 20.1 |
|  |  |  |  |  |  |  |  |  |  |  |  |  |
| 15 | 2018 | 140 | -17.8 | 10.7 | 3.39 |  |  | -18.2 | 8.2 | 3.87 |  |  |
| 16 | 2018 | 141 | -18.1 | 10.5 | 3.34 |  |  | -19.3 | 8.6 | 4.49 |  |  |
| 17 | 2018 | 140 | -18.5 | 10.3 | 3.60 |  |  | -19.7 | 8.0 | 4.71 |  |  |
| 18 | 2018 | 136 | -18.0 | 10.0 | 3.31 | 6.2 | 25.8 | -18.8 | 7.4 | 4.11 | 4.7 | 19.1 |
| 19 | 2018 | 139 | -17.5 | 10.9 | 3.18 |  |  | -19.2 | 8.3 | 4.85 |  |  |
| 20 | 2018 | 131 | -17.6 | 11.0 | 3.23 |  |  | -18.6 | 7.8 | 3.97 |  |  |
| 21 | 2018 | 137 | -18.1 | 10.4 | 3.27 |  |  | -18.5 | 7.5 | 3.91 |  |  |
| 22 | 2018 | 139 | -17.5 | 9.6 | 3.29 |  |  | -18.6 | 6.2 | 4.05 |  |  |
| 23 | 2018 | 133 | -17.4 | 10.9 | 3.21 |  |  | -18.1 | 8.0 | 3.85 |  |  |
| 24 | 2018 | 137 | -17.9 | 9.1 | 3.34 | 5.7 | 24.7 | -18.8 | 6.5 | 4.04 | 2.9 | 17.4 |
| 25 | 2018 | 141 | -18.6 | 9.6 | 3.60 |  |  | -18.6 | 7.0 | 4.00 |  |  |
| 26 | 2018 | 146 | -19.3 | 10.5 | 3.21 |  |  | -18.9 | 7.9 | 4.29 |  |  |
| 27 | 2018 | 144 | -17.6 | 12.7 | 3.93 | 9.0 | 28.3 | -18.2 | 9.2 | 3.92 | 5.5 | 20.3 |
| 28 | 2018 | 132 | -17.7 | 10.1 | 3.30 | 8.1 | 26.8 | -18.6 | 8.1 | 4.30 | 6.2 | 19.6 |
| 29 | 2018 | 146 | -18.7 | 10.1 | 3.71 |  |  | -18.6 | 8.5 | 4.09 |  |  |
| 30 | 2018 | 134 | -17.1 | 9.1 | 3.20 |  |  | -17.9 | 7.7 | 3.86 |  |  |
| 31 | 2018 | 145 | -17.9 | 9.3 | 3.37 | 6.9 | 25.7 | -18.2 | 8.3 | 4.00 | 5.7 | 19.6 |
| 32 | 2018 | 150 | -18.0 | 11.9 | 3.43 | 5.6 | 26.7 | -18.5 | 8.2 | 4.10 | 4.5 | 19.7 |
| 33 | 2018 | 132 | -18.3 | 9.7 | 3.44 | 6.7 | 25.7 | -18.6 | 7.8 | 4.04 | 4.1 | 18.5 |
| 34 | 2018 | 136 | -17.6 | 9.2 | 3.27 |  |  | -18.1 | 8.0 | 3.86 |  |  |
| 35 | 2018 | 138 | -17.8 | 10.0 | 3.15 |  |  | -18.5 | 7.8 | 3.86 |  |  |
| 36 | 2018 | 145 | -17.3 | 9.1 | 3.27 | 6.4 | 24.2 | -18.4 | 7.5 | 3.97 | 4.8 | 19.6 |
| 37 | 2018 | 135 | -17.6 | 9.7 | 3.19 | 5.4 | 25.2 | -18.8 | 7.2 | 4.21 | 3.7 | 18.9 |
| 38 | 2018 | 146 | -17.6 | 10.0 | 3.17 | 5.3 | 25.0 | -18.9 | 7.5 | 4.38 | 2.5 | 19.2 |
| 39 | 2018 | 133 | -18.0 | 9.1 | 3.47 |  |  | -18.6 | 7.9 | 4.28 |  |  |
| 40 | 2018 | 131 | -17.2 | 9.1 | 3.12 |  |  | -18.6 | 6.9 | 3.99 |  |  |
| 41 | 2018 | 139 | -17.7 | 10.0 | 3.14 |  |  | -18.3 | 7.9 | 4.06 |  |  |
| 42 | 2018 | 144 | -17.8 | 9.5 | 3.31 |  |  | -18.6 | 7.0 | 3.98 |  |  |
| 43 | 2018 | 137 | -18.1 | 9.6 | 3.47 |  |  | -19.1 | 8.1 | 4.79 |  |  |
| 44 | 2018 | 152 | -17.4 | 9.5 | 3.32 | 6.8 | 25.1 | -18.4 | 7.4 | 4.40 | 5.1 | 18.7 |
| 45 | 2018 | 146 | -17.6 | 10.1 | 3.25 | 4.4 | 24.0 | -18.7 | 7.9 | 4.43 | 3.0 | 18.8 |
| 46 | 2018 | 146 | -17.6 | 10.0 | 3.16 |  |  | -18.9 | 6.9 | 4.16 |  |  |
| 47 | 2018 | 143 | -17.5 | 10.0 | 3.18 |  |  | -18.6 | 8.1 | 4.14 |  |  |
| 48 | 2018 | 135 | -17.3 | 10.0 | 3.20 | 7.8 | 25.3 | -18.6 | 7.0 | 4.04 | 4.3 | 18.1 |
| 49 | 2018 | 128 | -17.8 | 9.5 | 3.35 |  |  | -18.3 | 7.7 | 3.96 |  |  |
| 50 | 2018 | 142 | -17.5 | 10.1 | 3.23 |  |  | -18.3 | 7.8 | 4.16 |  |  |
| 51 | 2018 | 139 | -17.6 | 9.5 | 3.19 | 7.4 | 24.7 | -18.5 | 8.0 | 4.06 | 3.5 | 18.5 |
| 52 | 2018 | 146 | -18.4 | 10.0 | 3.56 |  |  | -20.0 | 7.3 | 5.23 |  |  |
| 53 | 2018 | 130 | -17.8 | 9.5 | 3.25 |  |  | -18.6 | 8.1 | 4.23 |  |  |
| 54 | 2018 | 131 | -17.7 | 9.7 | 3.30 | 4.7 | 23.6 | -19.0 | 7.3 | 4.08 | 3.4 | 18.7 |
| 55 | 2018 | 138 | -17.7 | 9.9 | 3.21 |  |  | -18.7 | 7.1 | 3.99 |  |  |
| 56 | 2018 | 154 | -17.8 | 10.0 | 3.36 |  |  | -18.6 | 7.9 | 4.25 |  |  |
| 57 | 2018 | 134 | -17.4 | 9.4 | 3.23 |  |  | -18.7 | 7.6 | 4.39 |  |  |
| 58 | 2018 | 135.5 | -17.6 | 9.8 | 3.22 |  |  | -18.2 | 7.6 | 4.17 |  |  |
| 59 | 2018 | 138 | -17.5 | 10.7 | 3.26 |  |  | -19.1 | 7.2 | 4.29 |  |  |
| 60 | 2018 | 137 | -18.1 | 9.8 | 3.42 | 7.4 | 25.5 | -19.5 | 7.3 | 4.69 | 3.0 | 17.8 |
| 61 | 2018 | 146 | -18.6 | 10.2 | 3.75 |  |  | -18.9 | 8.2 | 4.17 |  |  |
| 62 | 2018 | 135 | -17.8 | 9.6 | 3.32 |  |  | -18.7 | 7.1 | 4.19 |  |  |
| 63 | 2018 | 152 | -17.8 | 10.3 | 3.28 |  |  | -19.1 | 8.2 | 4.39 |  |  |
| 64 | 2018 | 145 | -17.8 | 12.4 | 3.32 | 9.1 | 26.2 | -18.3 | 9.1 | 4.06 | 5.0 | 19.2 |
| 65 | 2018 | 160 | -17.4 | 10.2 | 3.17 |  |  | -19.2 | 7.0 | 4.57 |  |  |
| 66 | 2018 | 144 | -17.8 | 9.9 | 3.28 | 5.3 | 23.1 | -18.5 | 8.1 | 4.32 | 3.8 | 19.5 |
| 67 | 2018 | 154 | -17.9 | 12.9 | 3.34 | 7.7 | 28.6 | -17.9 | 10.9 | 3.73 | 6.8 | 21.4 |
| 68 | 2018 | 145 | -17.5 | 9.9 | 3.29 |  |  | -18.5 | 7.9 | 4.09 |  |  |
| 69 | 2018 | 134 | -17.3 | 9.6 | 3.22 | 5.6 | 23.4 | -18.6 | 7.5 | 4.19 | 3.8 | 19.0 |
| 70 | 2018 | 138 | -17.9 | 9.7 | 3.31 | 7.2 | 24.4 | -18.9 | 7.6 | 4.30 | 2.9 | 19.4 |
| 71 | 2018 | 152 | -17.5 | 9.9 | 3.29 |  |  | -18.2 | 7.5 | 4.22 |  |  |
| 72 | 2018 | 145 | -18.0 | 10.3 | 3.26 | 4.0 | 23.7 | -19.1 | 7.4 | 4.47 | 1.7 | 19.3 |
