# Supplementary material for: Yellowfin tuna (Thunnus albacares) foraging habitat and trophic position in the Gulf of Mexico based on intrinsic isotope tracers
Source: PLoS One. 2021 Feb 24;16(2):e0246082. doi: 10.1371/journal.pone.0246082 (PMC7904200; doi:10.1371/journal.pone.0246082)
Supplement: S2 Table — Cruise name, stations, coordinates in decimal degrees, and δ13C and δ15N values of zooplankton (fraction size 335–1000 μm). *Stations used as northern Gulf of Mexico (GM), †Stations used as central-southern GM. (DOCX) [file pone.0246082.s003.docx]

**S2 Table**. **Summary of zooplankton sampling cruises.** Cruise, stations, coordinates in decimal degrees, and δ^13^C and δ^15^N values of zooplankton (fraction size 335-1000 μm) are shown. *Correspond to stations used as northern Gulf of Mexico (GM), ^†^correspond to stations used as central-southern GM.

| **Cruise** | **Station ID** | **Latitude** | **Longitude** | **δ^13^C** | **δ ^15^N** |
| --- | --- | --- | --- | --- | --- |
| GOMECC-03 | E1^†^ | 26.004 | -86.004 | -19.1 | 1.2 |
| GOMECC-03 | E2^†^ | 26.656 | -85.004 | -20.5 | 2.4 |
| GOMECC-03 | E3* | 27.330 | -84.002 | -21.7 | 4.8 |
| GOMECC-03 | E4* | 27.775 | -83.332 | -20.5 | 4.9 |
| GOMECC-03 | E5* | 29.378 | -85.511 | -21.1 | 6.4 |
| GOMECC-03 | E6* | 29.027 | -85.790 | -21.0 | 7.9 |
| GOMECC-03 | E8* | 28.000 | -86.635 | -20.5 | 3.1 |
| GOMECC-03 | E9* | 27.581 | -90.004 | -20.3 | 3.5 |
| GOMECC-03 | E10* | 27.923 | -89.999 | -19.9 | 3.3 |
| GOMECC-03 | E11* | 28.498 | -90.001 | -21.1 | 8.9 |
| GOMECC-03 | E12* | 28.937 | -90.123 | -22.7 | 11.6 |
| GOMECC-03 | E13* | 27.813 | -93.840 | -22.0 | 7.2 |
| GOMECC-03 | E14* | 27.809 | -93.840 | -21.7 | 7.4 |
| GOMECC-03 | E15* | 27.815 | -93.848 | -22.3 | 7.3 |
| GOMECC-03 | E16* | 27.813 | -93.840 | -22.5 | 7.2 |
| GOMECC-03 | E17* | 28.090 | -95.001 | -20.1 | 5.3 |
| GOMECC-03 | E18* | 28.335 | -94.999 | -21.8 | 8.4 |
| GOMECC-03 | E19* | 28.669 | -94.997 | -22.7 | 9.4 |
| GOMECC-03 | E20* | 29.002 | -94.999 | -20.2 | 11.6 |
| GOMECC-03 | E21* | 25.878 | -96.802 | -21.9 | 4.7 |
| GOMECC-03 | E22^†^ | 25.880 | -96.323 | -20.0 | 1.7 |
| GOMECC-03 | E23^†^ | 25.881 | -95.830 | -19.4 | 2.0 |
| GOMECC-03 | E24^†^ | 25.878 | -94.672 | -19.6 | 2.0 |
| GOMECC-03 | E25^†^ | 22.274 | -97.546 | -21.4 | 3.2 |
| GOMECC-03 | E26^†^ | 22.269 | -97.358 | -20.0 | 2.6 |
| GOMECC-03 | E27^†^ | 22.267 | -96.763 | -20.4 | 1.7 |
| GOMECC-03 | E28^†^ | 22.268 | -94.993 | -20.6 | 2.3 |
| GOMECC-03 | E29^†^ | 25.046 | -88.011 | -19.6 | 2.2 |
| GOMECC-03 | E30^†^ | 24.394 | -87.990 | -21.1 | 2.2 |
| GOMECC-03 | E31^†^ | 23.772 | -87.997 | -21.3 | 2.7 |
| GOMECC-03 | E32^†^ | 21.509 | -95.602 | -19.2 | 4.0 |
| GOMECC-03 | E33^†^ | 21.450 | -91.564 | -19.1 | 4.3 |
| GOMECC-03 | E34^†^ | 21.736 | -92.315 | -20.9 | 3.3 |
| GOMECC-03 | E35^†^ | 21.502 | -92.541 | -20.6 | 3.2 |
| GOMECC-03 | E36^†^ | 20.735 | -94.750 | -20.1 | 3.2 |
| GOMECC-03 | E37^†^ | 20.019 | -93.762 | -19.9 | 4.1 |
| GOMECC-03 | E38^†^ | 19.173 | -93.299 | -21.9 | 4.5 |
| GOMECC-03 | E39^†^ | 18.834 | -93.065 | -21.0 | 5.2 |
| GOMECC-03 | E40^†^ | 21.590 | -86.497 | -19.0 | 5.0 |
| GOMECC-03 | E41^†^ | 21.636 | -86.232 | -19.6 | 3.3 |
| GOMECC-03 | E43 | 21.833 | -84.982 | -19.9 | 1.4 |
| GOMECC-03 | E44 | 23.268 | -80.616 | -19.4 | 2.3 |
| GOMECC-03 | E45 | 23.785 | -80.617 | -20.4 | 1.7 |
| GOMECC-03 | E46b | 24.338 | -80.578 | -19.3 | 2.2 |
| GOMECC-03 | E47 | 24.745 | -80.619 | -14.8 | 3.8 |
| GOMECC-03 | E48 | 26.986 | -80.004 | -17.8 | 3.9 |
| GOMECC-03 | E49 | 27.006 | -79.875 | -20.5 | 3.4 |
| GOMECC-03 | E50 | 26.988 | -79.610 | -20.2 | 3.4 |
| GOMECC-03 | E51 | 26.998 | -79.196 | -19.0 | 2.4 |
| XIXIMI-06 | A1^†^ | 24.881 | -95.515 | -20.3 | 1.6 |
| XIXIMI-06 | A2^†^ | 24.883 | -94.985 | -20.5 | 1.6 |
| XIXIMI-06 | A10^†^ | 24.937 | -87.068 | -19.2 | 1.5 |
| XIXIMI-06 | B11^†^ | 24.007 | -96.012 | -20.5 | 1.6 |
| XIXIMI-06 | B12^†^ | 23.996 | -95.086 | -21.1 | 1.6 |
| XIXIMI-06 | B13^†^ | 23.976 | -93.711 | -20.3 | 2.2 |
| XIXIMI-06 | B14^†^ | 24.056 | -92.318 | -17.3 | 1.6 |
| XIXIMI-06 | B15^†^ | 23.992 | -90.995 | -18.7 | 2.5 |
| XIXIMI-06 | B17^†^ | 24.010 | -89.008 | -20.4 | 2.7 |
| XIXIMI-06 | B18^†^ | 24.021 | -86.836 | -20.2 | 1.8 |
| XIXIMI-06 | C21^†^ | 22.999 | -95.500 | -20.2 | 0.9 |
| XIXIMI-06 | C22^†^ | 23.006 | -94.500 | -20.7 | 2.2 |
| XIXIMI-06 | C23^†^ | 22.977 | -93.024 | -20.7 | 1.6 |
| XIXIMI-06 | C24^†^ | 22.512 | -92.008 | -19.8 | 1.6 |
| XIXIMI-06 | C25^†^ | 22.997 | -91.021 | -19.6 | 3.1 |
| XIXIMI-06 | D26^†^ | 22.020 | -97.147 | -20.1 | 2.4 |
| XIXIMI-06 | D27^†^ | 22.000 | -96.001 | -19.3 | 2.1 |
| XIXIMI-06 | D28^†^ | 22.004 | -95.010 | -19.7 | 2.5 |
| XIXIMI-06 | D29^†^ | 22.009 | -94.026 | -20.4 | 3.6 |
| XIXIMI-06 | D30^†^ | 21.998 | -93.013 | -19.7 | 2.7 |
| XIXIMI-06 | E32^†^ | 22.540 | -88.001 | -20.3 | 2.6 |
| XIXIMI-06 | E33^†^ | 21.496 | -94.502 | -20.2 | 2.1 |
| XIXIMI-06 | E35^†^ | 21.995 | -92.913 | -21.6 | 3.2 |
| XIXIMI-06 | F37^†^ | 21.006 | -95.000 | -19.9 | 2.1 |
| XIXIMI-06 | F38^†^ | 21.008 | -93.997 | -20.2 | 3.2 |
| XIXIMI-06 | F39^†^ | 21.003 | -92.990 | -20.7 | 3.4 |
| XIXIMI-06 | G40^†^ | 20.503 | -96.007 | -20.8 | 2.9 |
| XIXIMI-06 | G42^†^ | 20.512 | -94.502 | -21.1 | 3.2 |
| XIXIMI-06 | G43^†^ | 20.511 | -93.510 | -20.0 | 3.9 |
| XIXIMI-06 | G44^†^ | 20.517 | -92.500 | -20.7 | 2.9 |
| XIXIMI-06 | H45a^†^ | 19.989 | -95.626 | -20.7 | 3.2 |
| XIXIMI-06 | H45b^†^ | 19.989 | -95.626 | -21.1 | 3.4 |
| XIXIMI-06 | H46x^†^ | 20.002 | -94.998 | -20.4 | 3.1 |
| XIXIMI-06 | H46y^†^ | 20.001 | -93.996 | -20.0 | 3.6 |
| XIXIMI-06 | H48^†^ | 20.012 | -93.011 | -21.3 | 5.5 |
| XIXIMI-06 | J49^†^ | 19.503 | -94.999 | -20.3 | 3.1 |
| XIXIMI-06 | Y2a^†^ | 21.602 | -86.348 | -19.5 | 2.7 |
| XIXIMI-06 | Y2b^†^ | 21.610 | -86.356 | -19.6 | 2.7 |
| XIXIMI-06 | Y3a^†^ | 21.662 | -86.245 | -18.7 | 2.7 |
| XIXIMI-06 | Y3b^†^ | 21.677 | -86.223 | -19.5 | 3.0 |
| XIXIMI-06 | Y6a^†^ | 21.676 | -86.059 | -19.5 | 2.4 |
| XIXIMI-06 | Y6b^†^ | 21.695 | -86.055 | -20.3 | 2.1 |
| XIXIMI-06 | Y7a^†^ | 21.718 | -85.950 | -19.9 | 2.4 |
| XIXIMI-06 | Y7b^†^ | 21.717 | -85.943 | -20.1 | 2.1 |
