# Supplementary material for: Yellowfin tuna (Thunnus albacares) foraging habitat and trophic position in the Gulf of Mexico based on intrinsic isotope tracers
Source: PLoS One. 2021 Feb 24;16(2):e0246082. doi: 10.1371/journal.pone.0246082 (PMC7904200; doi:10.1371/journal.pone.0246082)
Supplement: S3 Table — Estimates for yellowfin tuna caught in the central and southern Gulf of Mexico (GM). (DOCX) [file pone.0246082.s004.docx]

**S3 Table**. **Mean trophic position based on liver tissue.** Mean trophic position (TP) estimates for yellowfin tuna caught in the central and southern Gulf of Mexico (GM) based on liver tissues.

| **TP estimation method** | **Source** | **Organism and approach for estimations of TEFs or TDFs** | **Tissue** | **TEF (bulk) or TDF (CSIA-AA) (‰)** | **Calculated TP**  **Mean ± SD** | |
| --- | --- | --- | --- | --- | --- | --- |
|  |  |  |  |  | Northern GM | Central-southern GM |
| Bulk δ^15^N values | Madigan et al. [77] | Pacific bluefin tuna (*Thunnus orientalis*) held in captivity and fed with natural diet | Liver | 1.1 ± 0.6 | **5.7 ± 0.7** | **8.6 ± 0.7** |
| CSIA | Nuche-Pascual [85] | Carnivorous yellowtail fed an optimal protein diet in controlled feeding experiments | Liver | 4.0 ± 2.5 | **3.9 ± 0.2** | |

TP calculated using two approaches: (1) using YFT δ^15^N_bulk_ values and the northern GM and central-southern GM regional isotopic baselines with mean δ^15^N values of 6.0 ± 3.1‰ and 2.8 ± 1.0‰, respectively and (2) δ^15^N_Phe_ of YFT liver and zooplankton. TEFs for TP_bulk_ estimates and trophic discrimination factors (TDF =TEF_Glu-_TEF_Phe_) for TP_CSIA_ estimates derived from the literature. The SD for liver TEF was calculated as the propagated error of the analytical uncertainty of Phe and Glu.
