# Supplementary material for: Yellowfin tuna (Thunnus albacares) foraging habitat and trophic position in the Gulf of Mexico based on intrinsic isotope tracers
Source: PLoS One. 2021 Feb 24;16(2):e0246082. doi: 10.1371/journal.pone.0246082 (PMC7904200; doi:10.1371/journal.pone.0246082)
Supplement: S4 Table — Estimates based on stomach content analysis (SCA), bulk δ15N analysis, and CSIA-AA δ15N of muscle tissue. The standard deviation is presented when reported by the authors or when it could be calculated from the raw data. (DOCX) [file pone.0246082.s005.docx]

**S4 Table**. **Mean trophic position (TP) estimates for yellowfin tuna sampled throughout its distribution.**  Mean trophic position (TP) estimates for yellowfin tuna sampled throughout its distribution based on stomach content analysis (SCA), bulk δ^15^N analysis, and CSIA-AA δ^15^N of muscle tissue. The standard deviation is presented when reported by the authors or when it could be calculated from the raw data.

| **Reference** | **Location** | **Method** | **TP** |
| --- | --- | --- | --- |
| Olson et al. (2010) | Pelagic eastern Pacific Ocean | SCA | 3.7 ± 0.3 |
| Bradley et al. (2015) | Eastern Tropical Pacific |  | 4.3 ± 0.7 |
| Pethybridge et al. (2018) | Global mean | Bulk δ^15^N | 4.7 ± 0.9 |
| Houssard et al. (2017) | Western and Central Pacific Ocean |  | 4.3 ± 0.1 |
| Varela et al. (2017) | Ecuadorian waters |  | 3.7 |
| Weng et al. (2015) | Southwestern Taiwan |  | 4.5 ± 0.6 |
| Logan & Lutcavage (2013) | Central North Atlantic Ocean |  | 4.5 ± 0.3 |
| Olson et al. (2010) | Pelagic eastern Pacific Ocean |  | 4.7 ± 0.05 |
| Rooker et al. (2006) | Northwestern Gulf of Mexico |  | 3.3 |
| Houssard et al. (2017) | Western and Central Pacific Ocean | CSIA AA δ^15^N | 4.1 ± 0.7 |
| Popp et al. (2007) | Oriental Pacific Ocean |  | 4.4 |
