# Supplementary material for: Yellowfin tuna (Thunnus albacares) foraging habitat and trophic position in the Gulf of Mexico based on intrinsic isotope tracers
Source: PLoS One. 2021 Feb 24;16(2):e0246082. doi: 10.1371/journal.pone.0246082 (PMC7904200; doi:10.1371/journal.pone.0246082)
Supplement: S5 Table — Estimates using five literature derived TEFs using bulk δ15N analyses (SIA) and two baselines and TP derived from δ15N values of Glu and Phe (CSIA). *indicates a significant relationship between curved fork length (CFL) and trophic position (TP). (DOCX) [file pone.0246082.s006.docx]

**S5 Table. Pearson's correlation between curved fork length (CFL) and each trophic position (TP).** Pearson’s correlation between curved fork length (CFL) and each trophic position (TP) estimates using five literature derived TEFs using bulk δ^15^N analyses (SIA) and two baselines and TP derived from δ^15^N values of Glu and Phe (CSIA). References for TEFs and TDFs following order of appearance in *Results*, Table 2. *indicates a significant relationship between curved furcal length (CFL) and trophic position (TP).

|  | | CFL | |
| --- | --- | --- | --- |
| TEF/TDF | Method | Northern GM | Central-southern GM |
| 1.9 | TPSIA1 | r= 0.33  p=0.005* | r= 0.34  p= 0.004* |
| 1.1 | TPSIA2 | r= 0.34  p= 0.003* | r= 0.34  p= 0.003* |
| 6.3 | TPCSIA1 | r= -0.05  p= 0.792 | |
| 5.7 | TPCSIA2 | r= -0.07  p= 0.679 | |
| 4.0 | TPCSIA3 | r= 0.17  p= 0.317 | |
