# Supplementary material for: Yellowfin tuna (Thunnus albacares) foraging habitat and trophic position in the Gulf of Mexico based on intrinsic isotope tracers
Source: PLoS One. 2021 Feb 24;16(2):e0246082. doi: 10.1371/journal.pone.0246082 (PMC7904200; doi:10.1371/journal.pone.0246082)
Supplement: S6 Table — Stations classified as northern GM and central-southern GM, and outside the GM (OGM). δ15N values of zooplankton (fraction size >2000 μm) of the canonical source amino acid (Phe). (DOCX) [file pone.0246082.s007.docx]

**S6 Table**. **Zooplankton isotopic composition by regions of the Gulf of Mexico (GM).** Stations classified as northern Gulf of Mexico (GM) and central-Southern GM, and outside the Gulf of Mexico (OGM) δ^15^N values of zooplankton (fraction size >2000 μm) of Phe.

|  | **Stations** | **Phe** | **Glu** |
| --- | --- | --- | --- |
| Northern GM | E5+E6 | 3.2 | 19.6 |
|  | E11 | 4.2 | 18.5 |
|  | E12 | 6.6 | 22.4 |
|  | E19 | 5.4 | 20.3 |
|  | E20 | 8.5 | 22.9 |
|  | E21 | 4.2 | 15.8 |
| Central-South GM | E22+E23 | -0.9 | 11.1 |
|  | E25+E26 | -1.6 | 13.5 |
|  | A1+2-B11+12 | -1.1 | 14.0 |
|  | B14+B15 | -1.6 | 14.5 |
|  | B17 | -0.3 | 15.4 |
|  | E32+E33 | -0.4 | 13.4 |
|  | G43 | -0.7 | 15.1 |
|  | G40 | -2.0 | 13.7 |
|  | E34 | 1.9 | 16.0 |
|  | E38+E39 | 1.3 | 15.7 |
|  | Y6a+Y6b+Y7b | -0.9 | 14.6 |
|  | Y2a+Y2b+Y3a | -1.1 | 16.1 |
|  | E43 | -0.2 | 13.2 |
|  | E45 | -1.0 | 14.7 |
| Off-Florida | E48+E50 | 0.0 | 17.7 |
|  | E49+E51 | 0.2 | 15.0 |
